# Algal Polysaccharides-Based Hydrogels: Extraction, Synthesis, Characterization, and Applications

**DOI:** 10.3390/md20050306

**Published:** 2022-04-29

**Authors:** Jianan Lin, Guangling Jiao, Azadeh Kermanshahi-pour

**Affiliations:** 1 Biorefining and Remediation Laboratory, Department of Process Engineering and Applied Science, Dalhousie University, 1360 Barrington St., Halifax, NS B3J 1Z1, Canada; jianan.lin@dal.ca; 2 AKSO Marine Biotech Inc., Suite 3, 1697 Brunswick St., Halifax, NS B3J 2G3, Canada; guangling@akso.ca

**Keywords:** algal polysaccharides, hydrogels, polysaccharides-based hydrogels, natural polymers

## Abstract

Hydrogels are three-dimensional crosslinked hydrophilic polymer networks with great potential in drug delivery, tissue engineering, wound dressing, agrochemicals application, food packaging, and cosmetics. However, conventional synthetic polymer hydrogels may be hazardous and have poor biocompatibility and biodegradability. Algal polysaccharides are abundant natural products with biocompatible and biodegradable properties. Polysaccharides and their derivatives also possess unique features such as physicochemical properties, hydrophilicity, mechanical strength, and tunable functionality. As such, algal polysaccharides have been widely exploited as building blocks in the fabrication of polysaccharide-based hydrogels through physical and/or chemical crosslinking. In this review, we discuss the extraction and characterization of polysaccharides derived from algae. This review focuses on recent advances in synthesis and applications of algal polysaccharides-based hydrogels. Additionally, we discuss the techno-economic analyses of chitosan and acrylic acid-based hydrogels, drawing attention to the importance of such analyses for hydrogels. Finally, the future prospects of algal polysaccharides-based hydrogels are outlined.

## 1. Introduction

Hydrogels are three-dimensional, crosslinked, hydrophilic polymeric networks capable of swelling in water and absorbing a considerable amount of water [1]. Chemical or physical crosslinks between the polymer chains prevent the polymeric networks from dissolving [2]. The concept of hydrogels was first put forward in 1894 when the term was used to describe a colloidal gel of inorganic salts [3]. In 1960, Wichterle and Lim published a landmark article in the scientific journal, *Nature*, in which synthetic poly(2-hydroxyethyl methacrylate) gels were designed as soft contact lenses [4]. These promising materials have been widely applied in the fields of biotechnology, including drug delivery [1,2], tissue engineering [5,6], wound dressing [7,8], agrochemicals application [9,10], separation [11,12], food packaging [13,14], and cosmetics and personal care products [15,16]. The hydrophilic polymer structures can be designed using physical and/or chemical crosslinking methods. Generally, hydrogels can be chemically crosslinked via crosslinker addition [17,18,19], photo- or radiation-initiated free radical polymerization [20,21,22], or enzyme-assisted reaction [23,24]. They can also be produced based on noncovalent interactions, such as electrostatic interaction, hydrogen bonding, van der Waals force, host-guest interaction, and hydrophobic interaction [25,26,27,28].

According to the source of materials, hydrogels can be divided into natural and synthetic hydrogels. Up to now, a variety of hydrogels with unique functions have been obtained from renewable natural polymers, among which, hydrogels derived from algal polysaccharides have attracted tremendous interest due to their outstanding biocompatibility and biodegradability. In recent decades, several polysaccharides—alginate, agarose, carrageenan, fucoidan, ulvan, laminarin, porphyrin, starch, and cellulose—have been extracted from marine seaweeds, including red algae [29,30,31,32], brown algae [33,34,35,36], and green algae [37,38]. As the research further develops, algal polysaccharides have been identified as important bioactive natural compounds with various well-documented benefits: anti-wrinkle, UV protection, antioxidant, immunomodulatory, anti-inflammatory, antibacterial and antiviral, and antiallergic qualities [39,40]. 

Algae are regarded as the most abundant and promising natural resources for the next generation of biorefineries. With the increasing growth of the algae industry and the development of algae biorefinery, the need for high-value applications of algae-extracted biopolymers is increasing. In this review, we present commonly used methods for the extraction and characterization of algal polysaccharides and further discuss the gelation mechanism and hydrogel preparation methods, as well as hydrogel characterization methods. This review also extends to discussing recent advances in promising application potentials of algal polysaccharides-based hydrogels. Additionally, techno-economic analyses of polysaccharides-based hydrogels (e.g., chitosan hydrogels) and a hydrogel based on acrylic acid, as well as their limitations, are discussed. Finally, we outline the prospect of algal polysaccharides-based hydrogels with respect to extraction, synthesis, and applications perspectives. 

## 2. Algae-Based Polysaccharides Extraction

**Figure 1:**
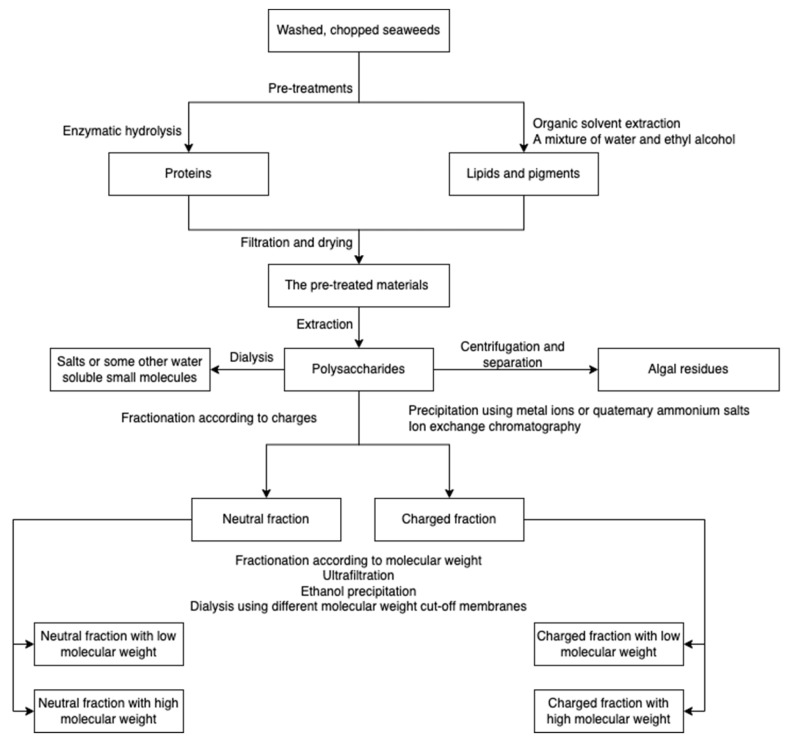
A schematic overview of algal polysaccharide extraction, purification, and fractionation.

Major algal polysaccharides produced at the industrial scale include agar, alginate, carrageenan, starch, and cellulose, among which, alginate, carrageenan, and agarose have been commercially exploited from seaweeds [41]. However, the production processes vary depending on the polysaccharide type. For example, highly branched laminarin can be extracted in both cold and hot acidic conditions, while ulvan and fucoidan can be extracted in only hot aqueous environments [42,43,44,45]. The composition and structure of polysaccharides vary depending on the species, collection sites, harvesting season, and water quality [46,47,48,49,50]. The conditions of extraction and purification processes influence the polysaccharide properties [51,52] and the resulting polysaccharide-derived hydrogels. However, the effect of polysaccharide structure and composition on hydrogel properties has not been widely studied due to the fact that for the synthesis of hydrogel, purchased algal polysaccharides are often used rather than obtaining the polysaccharides from extraction and purification [7,53,54]. A schematic overview for algal polysaccharide extraction is presented in Figure 1. Prior to polysaccharides extraction, seaweeds are usually pre-treated using organic solvents and enzymatic hydrolysis to remove non-targets/impurities. The resulting raw polysaccharides are further purified with dialysis, bleaching, etc. [34,55]. 

### 2.1. Alginate

**Figure 2:**
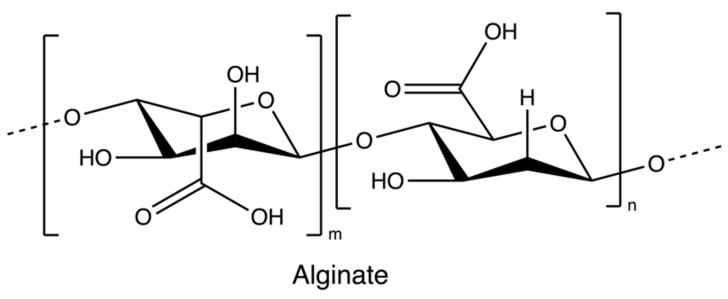
The chemical structure of Alginate.

Alginate (Figure 2) is an anionic polymer, first discovered in the 1880s, and industrial production started in 1929 [56]. It is mainly isolated from brown seaweed species, such as *Ascophyllum nodosum*, *Laminaria japonica*, *Laminaria hyperborean*, *Ecklonia maxima*, *Laminaria digitata*, and *Macrocystis pyrifera*. Its molecular structure comprises the copolymeric blocks of β-d-mannuronic acid and α-L-guluronic acid. Alginates usually present in a salt or acid form; the salt form plays a major role in the cell wall formation process of brown seaweed species. The acid form is known as alginic acid, which is present in the intercellular matrix material of brown algae as raw gels. The gels of alginate comprise salts of alginate with metal cations found in seawater, such as sodium ions [56,57].

**Figure 3:**
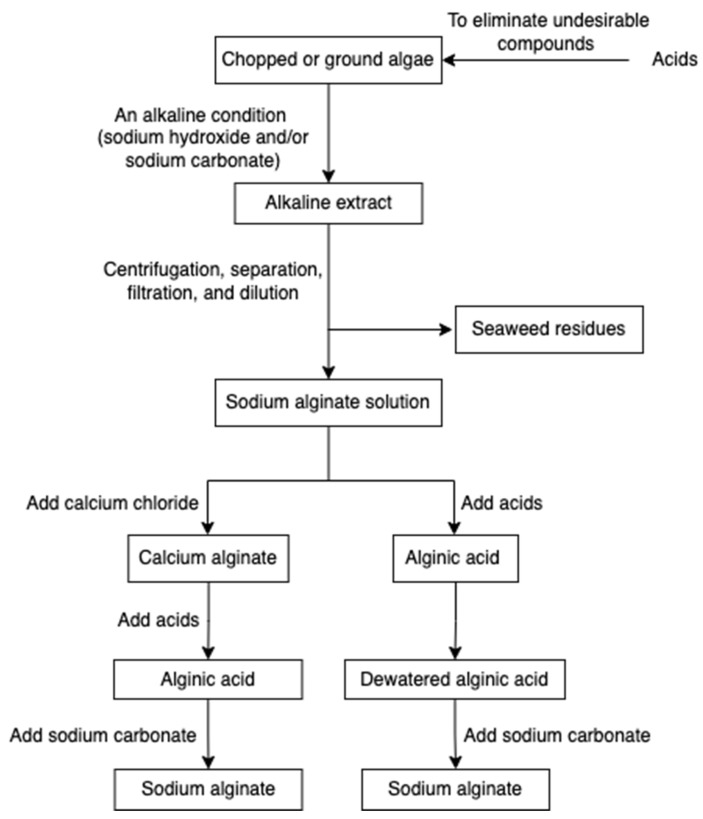
The scheme of alginate extraction.

The alginate extraction process involves several steps (Figure 3) [36,57,58,59]. Chopped or ground algae materials are treated with acids to eliminate undesirable compounds through the proton exchanging reaction. Subsequently, an alkaline condition (sodium hydroxide and/or sodium carbonate) is applied to neutralize the alginic acid, and hence insoluble alginic acids are converted into a sodium alginate solution. To remove seaweed residues, centrifugation followed by separation, filtration, and dilution are employed. The filtrated extract is then directly acidified to form alginic acid or undergoes an intermediate step of gelling as calcium alginate before conversion. The alginic acid and calcium alginate can also be further converted into sodium alginate.

Some innovative methods are also employed to extract alginate from brown seaweeds, such as the subcritical water hydrolysis extraction [33,60,61] and the microwave-assisted extraction [62,63], but those methods still remain in the lab stage.

### 2.2. Carrageenan

**Figure 4:**
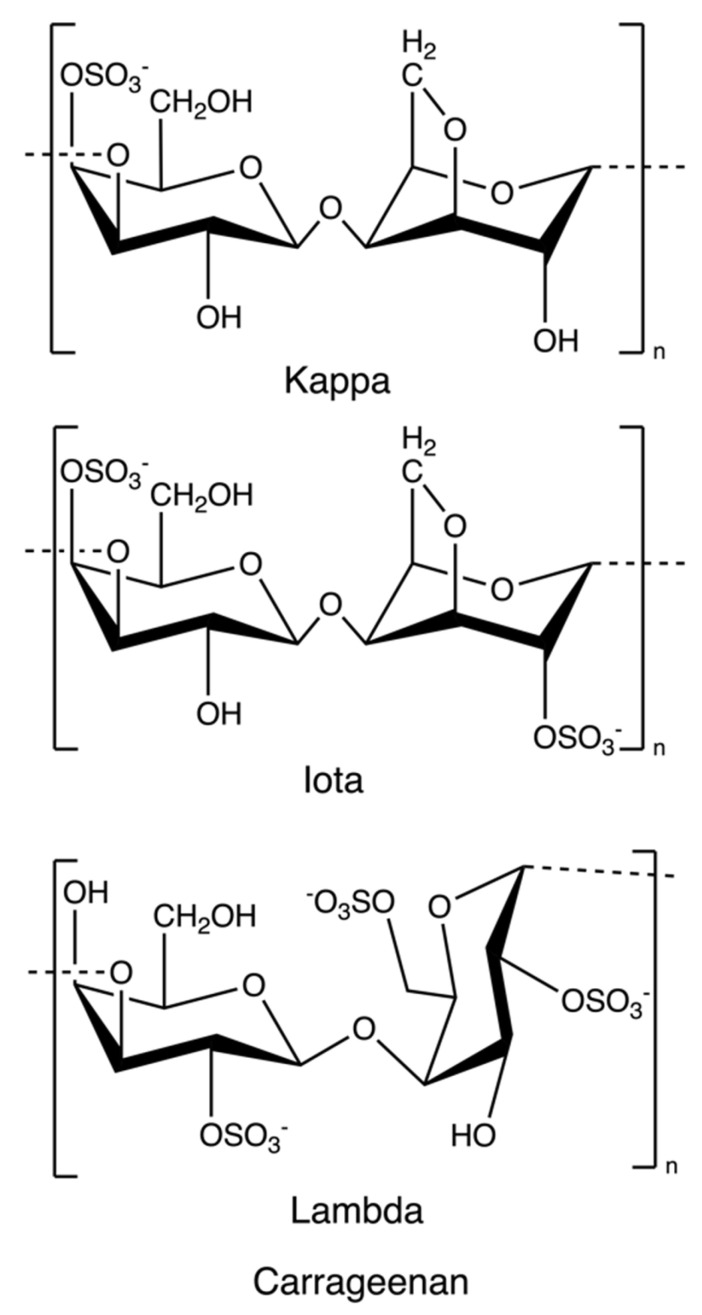
The chemical structure of kappa-, iota, and lambda-carrageenan.

Carrageenan is an anionic, sulphated polysaccharide consisting of alternating long linear chains of (1 → 3)-β-d-galactose and (1 → 4)-3, 6-anhydro-α-d-galactose (3, 6-AG) or (1 → 4)-α-d-galactose with ester sulphates (15–40%) [41]. These novel renewable natural polymers originate from the marine red algae family. They can be sorted into six basic forms depending on their source, solubility, and sulphate content: kappa, iota, lambda, mu, nu, and theta; among which, kappa, iota, and lambda (Figure 4) are commonly used as materials for hydrogel preparation due to their viscoelastic and gelling properties [64]. Currently, most carrageenans are extracted from *Kappaphycus alvarezii* and *Eucheuma denticulatum* [41], but some of them are still isolated from *Chondrus crispus* [65]. Original carrageenan, also known as refined carrageenan and filtered carrageenan, is used as a material for hydrogel production. 

**Figure 5:**
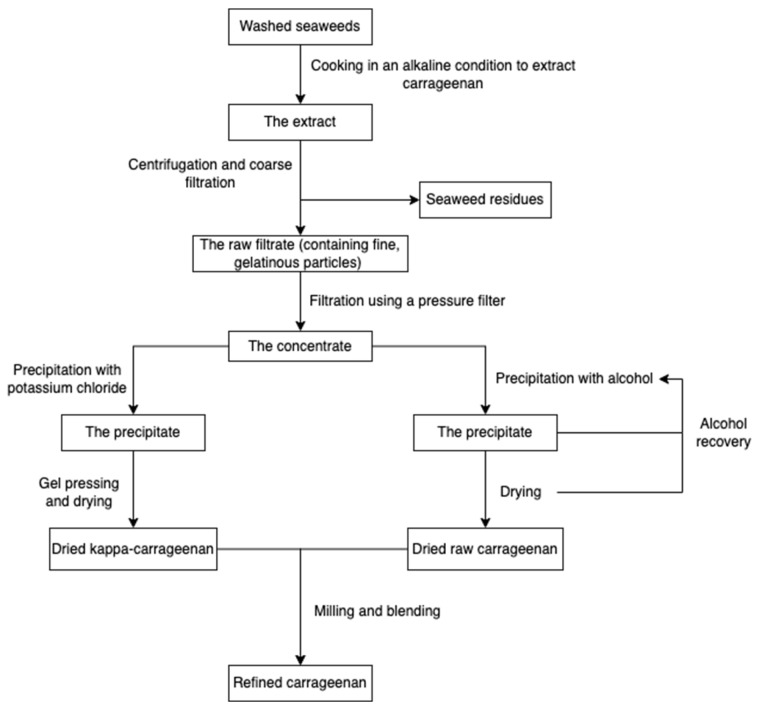
The scheme of carrageenan extraction.

The production procedure (Figure 5) started with cooking raw red seaweeds in alkaline conditions (e.g., sodium hydroxide) to increase the 3, 6-AG content and then extract carrageenans. After extraction, seaweed residues are filtered to obtain a concentrated polysaccharide solution. The polysaccharide solution is precipitated with isopropanol until acquiring fibrous coagulum, followed by separation, pressing, washing, drying, and milling. The alcohol-precipitation method is suitable for all carrageenans, while the gel method involving potassium chloride is usually used for the kappa-carrageenan extraction [55,65]. Carrageenans can also be extracted using eco-friendly technologies, such as microwave-assisted extraction [66,67] and the deep eutectic solvents method [68].

### 2.3. Agarose

**Figure 6:**
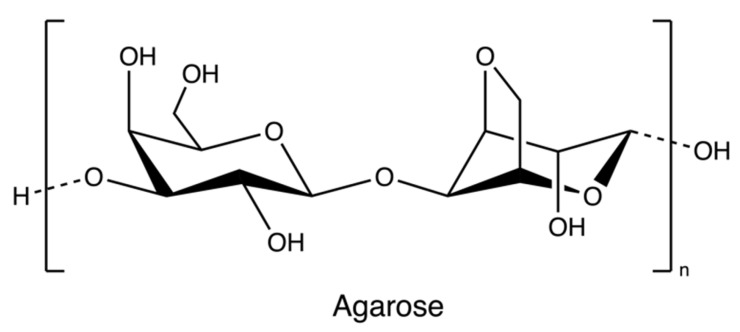
The chemical structure of agarose.

Agarose (Figure 6), a marine-based linear polysaccharide, is naturally derived from red algae, such as species of *Gelidium* and *Gracilaria*. Its gelling component, agarose, is an alternating copolymer of (1 → 3)-β-d-galactose and (1 → 4)-3,6-anhydro-α-L-galactose residues [69].

**Figure 7:**
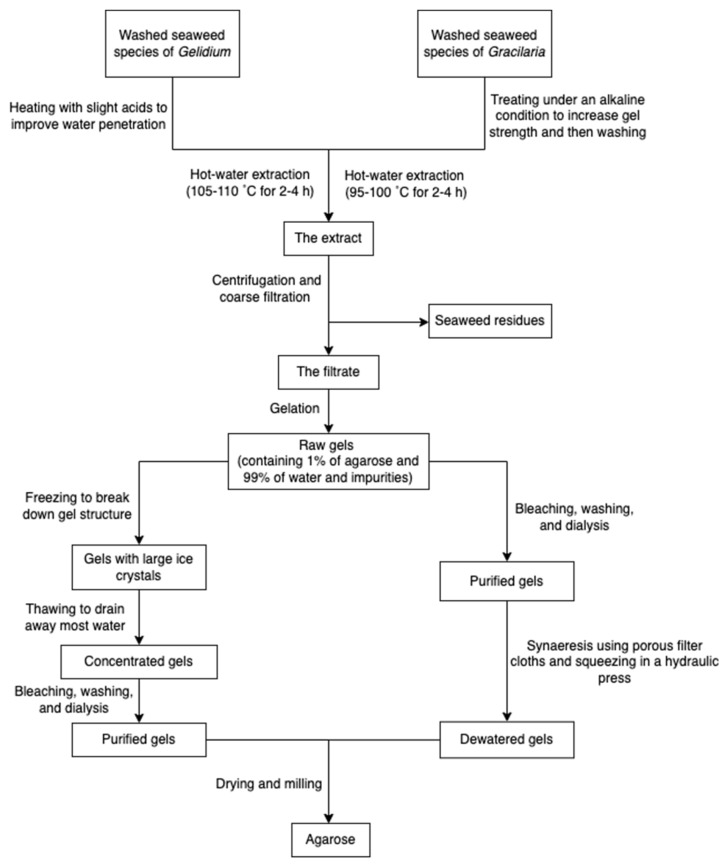
The scheme of agarose extraction.

The process of production of agarose varies from species. Species of *Gelidium* are usually pre-treated under heat with slight acids to improve water penetration, while species of *Gracilaria* are treated with alkali to increase the 3,6-AG content. The extraction processes (Figure 7) all rely on hot water extraction. After hot water extraction, the extract is filtered to remove seaweed residues. The agar filtrate is sequentially cooled to form a gel. The resulting gel may be treated with bleach to discolor it, washed to remove the bleach, and soaked in water to remove salts through osmosis. Because the gel primarily consists of water and impurities (about 99%), it is usually dried through the freezing/thawing process and pressing. However, compared with synaeresis using a porous filter and a hydraulic press, the freeze-thaw process is relatively expensive due to capital cost and energy consumption. Dewatered agar gel is further dried and milled [55]. Furthermore, to obtain pure agar, agaropectin can be removed through precipitation with poly(ethylene glycol) [70]. Researchers also used ionic liquids (solvents that contain only ions and have a melting point below 100 °C, such as choline acetate) as a medium to extract agarose from red algae [29].

### 2.4. Fucoidan

**Figure 8:**
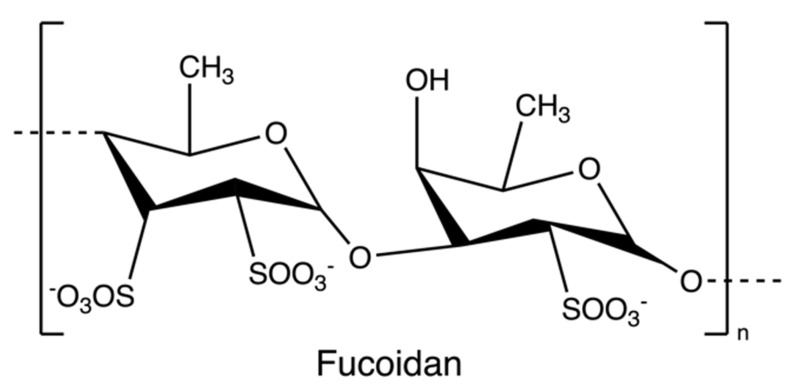
The chemical structure of fucoidan.

Fucoidans (Figure 8) are sulphated polysaccharides from the cell wall matrix of brown algae, which are comprised of α-1,3 and α-1,4 linked units of fucose with sulphate esters at positions 2, 3, and/or 4. However, the structure and sulfation pattern of the sugar backbone of these polysaccharides are species-specific [71].

**Figure 9:**
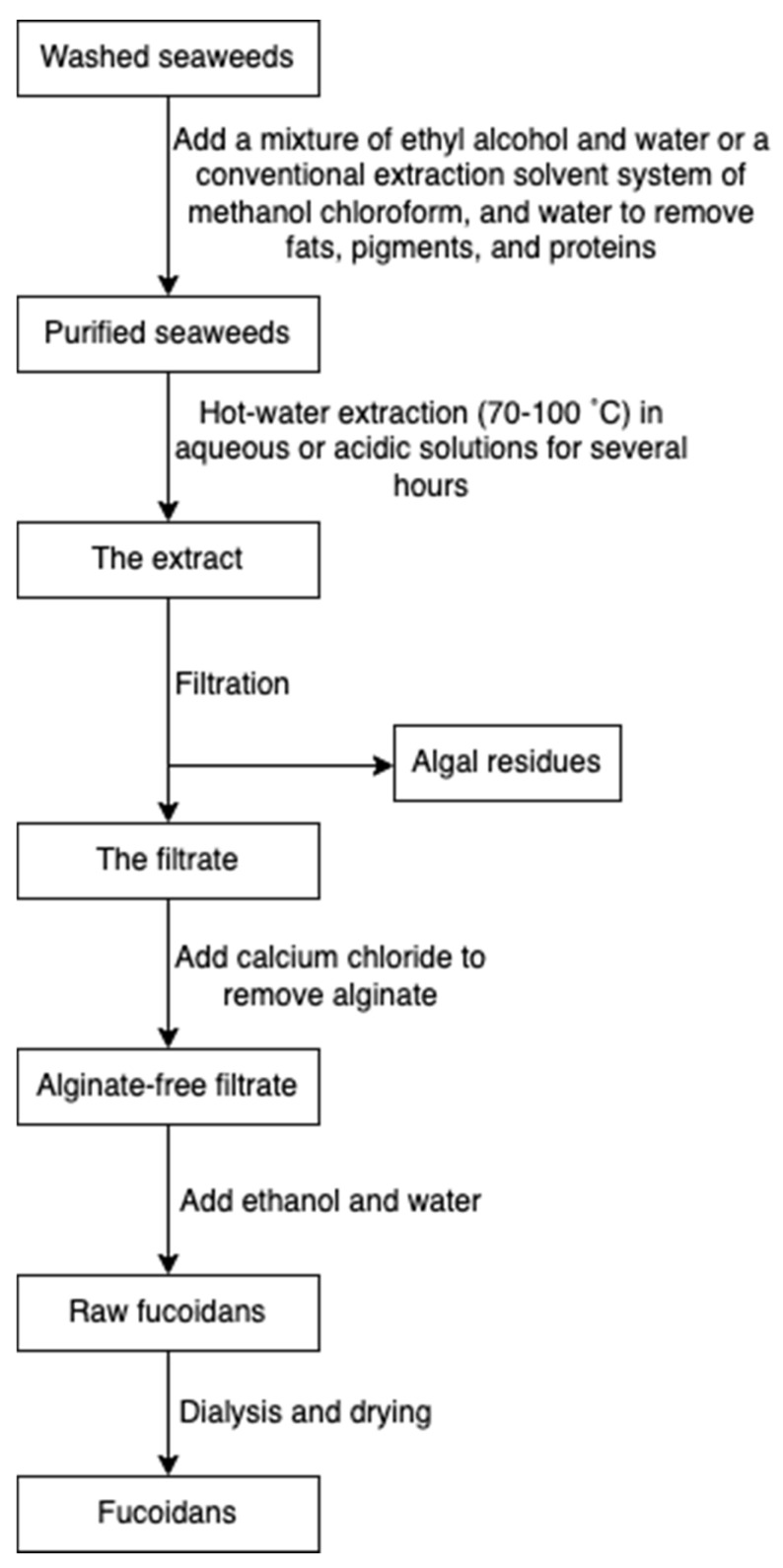
The scheme of fucoidan extraction.

Fucoidan is commonly extracted by cooking algal raw materials in hot aqueous or acidic solutions for several hours at 70 to 100 °C [45]. Prior to hot water extraction, fats, pigments, and proteins are firstly extracted with a mixture of ethyl alcohol and water or a conventional extraction solvent system of methanol, chloroform, and water. The pre-treatment step prevents the coextraction of other algal compounds [71]. Following hot water extraction, algae residues are filtered, and subsequently, calcium chloride is added to remove alginate. The mixture is re-extracted with ethanol and water to obtain fucoidans, followed by dialysis and drying (Figure 9) [34]. Some novel extraction procedures, such as microwave-assisted extraction [72] and enzyme-assisted extraction [73], have also been reported for fucoidan extraction.

### 2.5. Ulvan

**Figure 10:**
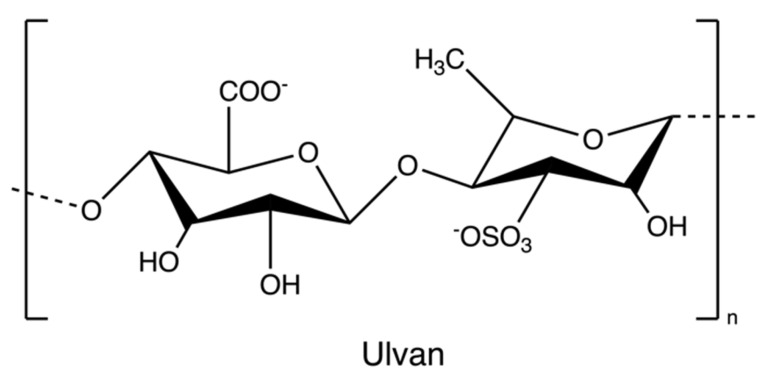
The chemical structure of ulvan.

**Figure 11:**
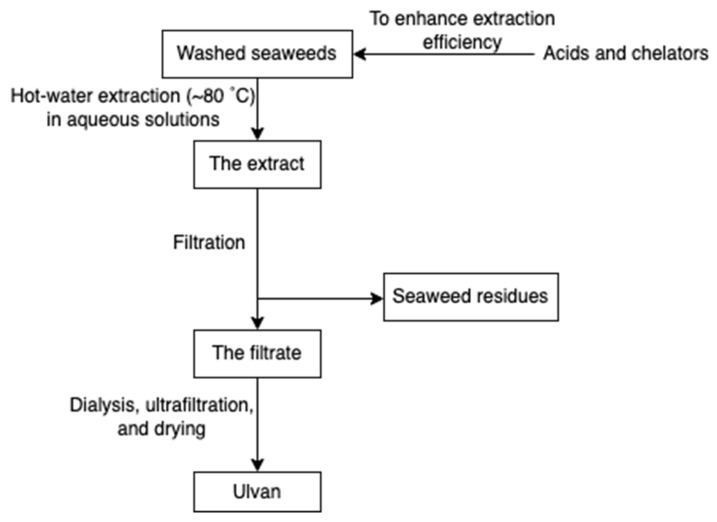
The scheme of ulvan extraction.

Ulvan (Figure 10) is a gelling sulphated polysaccharide from the species of green algae *Ulva*. It comprises sulphated rhamnose, uronic acids (glucuronic acid and iduronic acid), and xylose. The extraction yields and product quality are highly associated with the extraction and purification processes, pre-treatment, biomass sources, and storage [44]. Similarly, the solubility of ulvan in aqueous solutions can be improved by extraction at high temperatures over 80 °C but lower than the boiling point of the water (avoiding degradation) [43,44]. However, hot water extraction may result in low extraction yields due to the interaction between ulvan and algal cell wall components [37]. Therefore, chelators and acids are employed to overcome the structural integrity of the plant cell wall, hence increasing the extraction efficiency of ulvan. After extraction, dialysis and ultrafiltration are used to remove excess salts and small molecules in the ulvan extracts (Figure 11) [44]. Microwave-assisted extraction is an alternative to the conventional extraction of ulvan [43].

### 2.6. Laminarin

**Figure 12:**
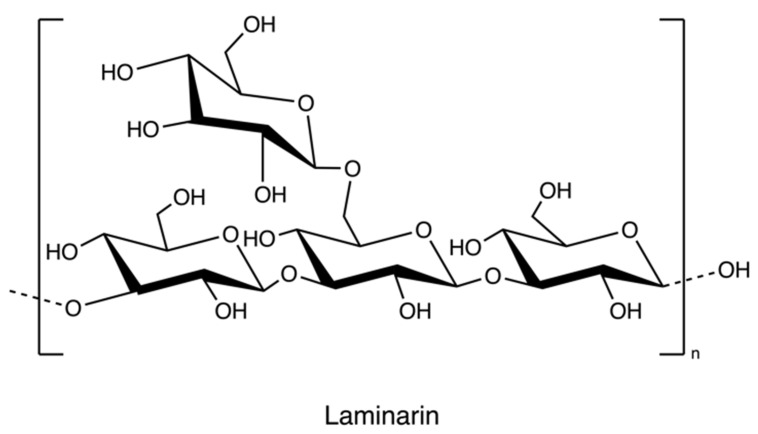
The chemical structure of laminarin.

Laminarin (Figure 12), a storage polysaccharide, is isolated from brown algae *Laminaria* and *Saccharina species*. It is a class of low molecular weight β-glucans. The structure includes (1,3)-β-d-glucopyranose residues with some 6-O-branching in the main chain and some β-(1,6)-intrachain links. Notably, it has been reported that laminarin content is up to 35% of brown algae on a dry weight basis [74].

**Figure 13:**
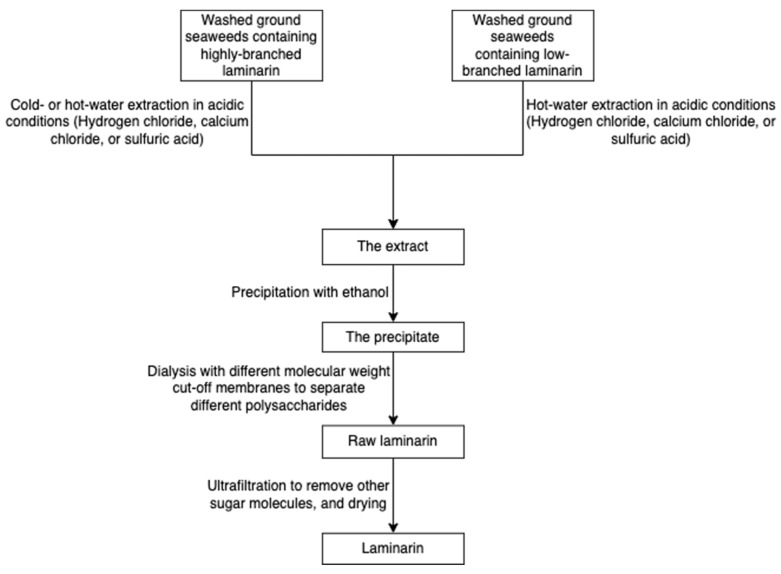
The scheme of laminarin extraction.

The solubility of laminarin depends on the level of branching. Highly branched laminarin is soluble in both cold water and hot water, while laminarin with a low branching level is soluble only in hot water [42]. In contrast to other gelling polysaccharides such as alginate and agarose, laminarin does not have thickening and gelling properties [75]. The general process of laminarin extraction at the lab level incorporates grinding, precipitation in an acid or basic medium, ultrafiltration, and dialysis (Figure 13) [76]. Because the molecular weight of laminarin is relatively lower than other polysaccharides in seaweeds, researchers employed dialysis coupled with molecular weight selection membranes to separate different polysaccharides [77]. A greener technique, ultrasound-assisted extraction, has been used to recover laminarin from Irish brown seaweeds [35]. 

### 2.7. Porphyran

**Figure 14:**
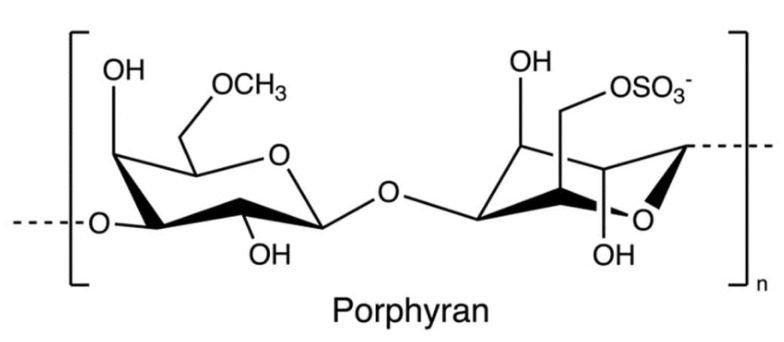
The chemical structure of porphyran.

**Figure 15:**
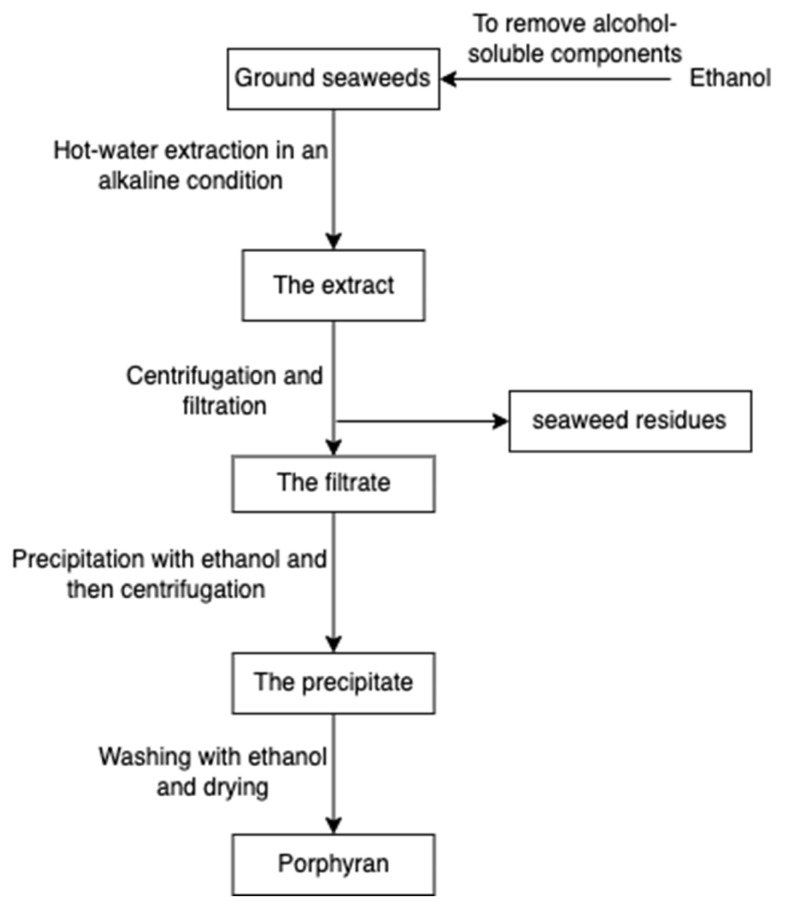
The scheme of porphyran extraction.

Porphyran (Figure 14), a sulphated polysaccharide from the red algae of *Porphyra yezoensis*, contains residues of d-galactose, L-galactose, 3, 6-anhydro-l-galactose, 6-O-methyl-d-galactose, and ester sulphate. The structure of porphyrin is similar to that of carrageenan and agarose, so the extraction process is comparable (Figure 15); for example, pre-treatment with alkali (e.g., sodium hydroxide) can increase 3, 6-AG content enhancing the gelation property [78]. Furthermore, ethanol is added to remove alcohol-soluble components in the pre-treatment [79].

### 2.8. Starch and Cellulose

**Figure 16:**
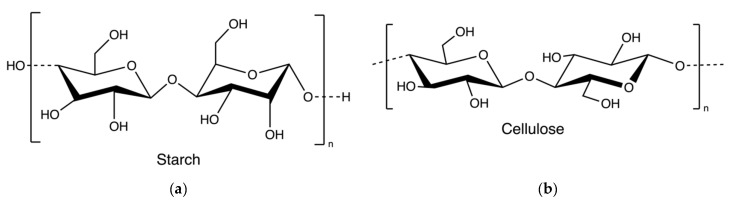
The chemical structure of starch (**a**) and cellulose (**b**).

**Figure 17:**
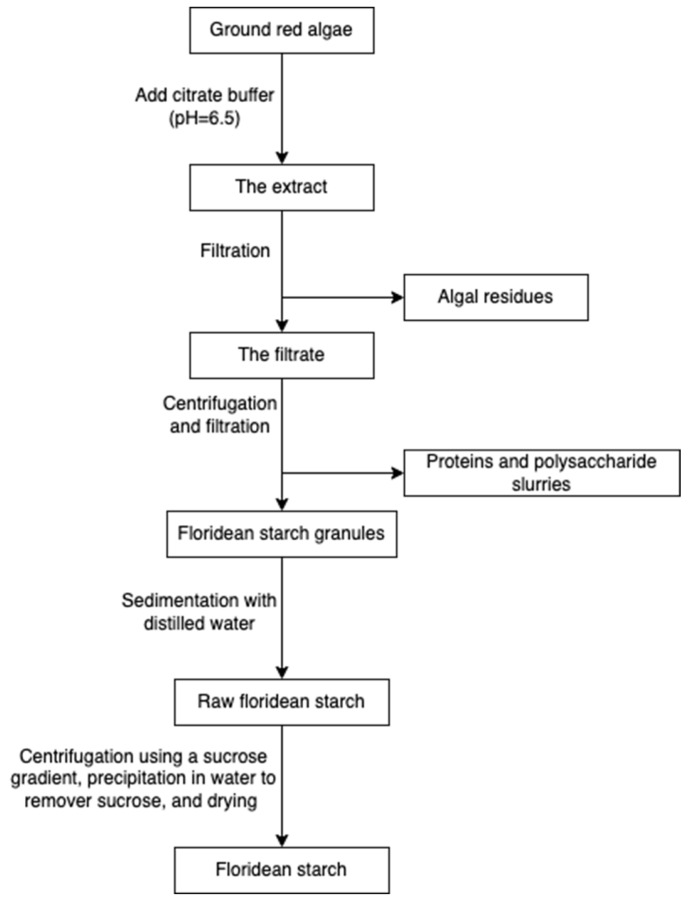
The scheme of starch extraction.

Starch and cellulose (Figure 16) are two common polysaccharides from plants, while they can also be found in algae. Floridean starch, isolated from red algae class *Florideophyceae*, is an α-1,4-glucosidic linked glucose homopolymer with α-1,6-branches functioning as carbon and energy reserve in the cells. Over 80% of the cell volume of red algae is filled with floridean starch. The extraction process involves citrate buffer treatment, filtration and separation, purification by sedimentation, centrifugation using a sucrose gradient, and precipitation in water (Figure 17) [30].

**Figure 18:**
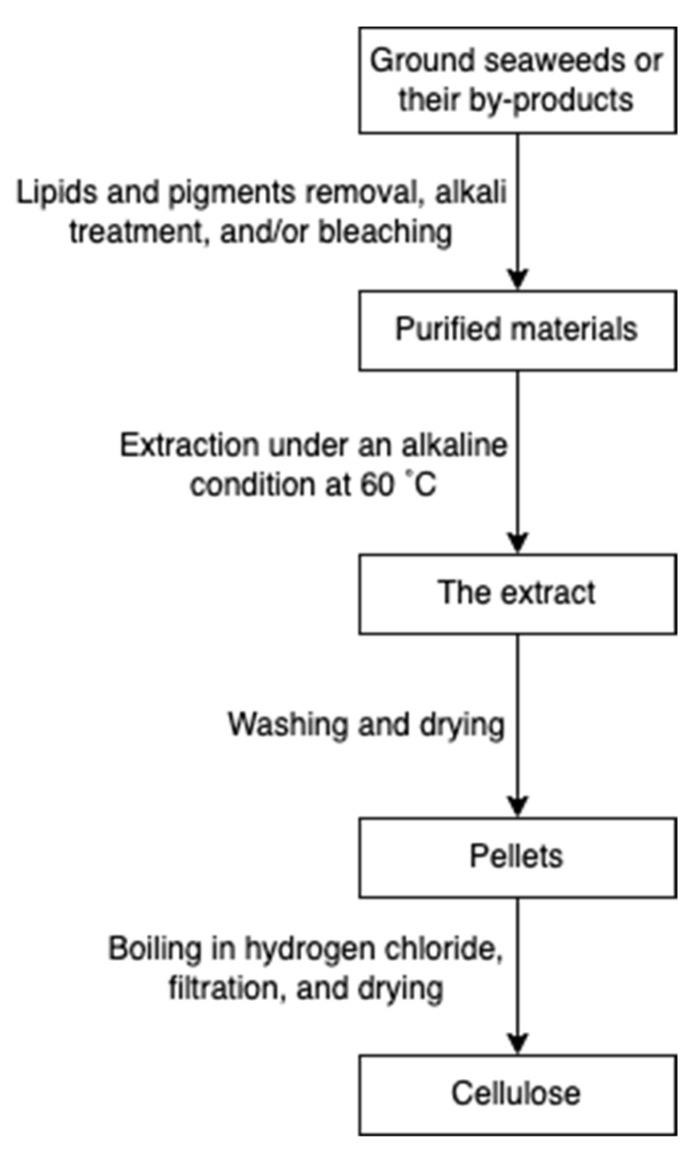
The scheme of cellulose extraction.

Cellulose is widely spread in green algae [38], brown algae [80], and red algae [31]. Particularly, it can be extracted from the by-products of the industrial extraction processes of algal polysaccharides [31,81]. To remove non-target compounds, the cellulose-rich wastes from algae are purified through lipids and pigments removal, alkali treatment, and/or bleaching steps (Figure 18). The purified materials are extracted with sodium hydroxide at 60 °C overnight [82]. Additionally, hydrochloric acid treatments can be added to increase the purity [81]. The purified cellulose can be further processed into hydrogel-forming derivates, such as cellulose nanofibers or nanocrystals [65].

## 3. Characterization of Algal Polysaccharides

Unlike synthetic polymers, which are prepared by a bottom-up approach (from monomers to polymers), natural polymers, such as polysaccharides from algae, are obtained through a top-down method. The complexity of the biomatrices may result in the extract containing impurities, such as proteins, lipids, inorganics, and other organic compounds [65]. Thus, it is necessary to determine the chemical composition and structure of the extracted algal polysaccharides. 

The properties of polysaccharides depend on their monomer composition. In order to analyze the monomer units eliminating the effects of the polymer structure, acid hydrolysis is used to decompose polysaccharides into monomeric sugars [83,84]. The monomeric sugars are analyzed and quantified by gas chromatography with mass spectrometry or a flame ionization detector (GC-MS/FID) [85,86,87], high-performance anion-exchange chromatography with a pulsed amperometric detector (HPAEC-PAD) [84], high-performance liquid chromatography with ultraviolet-visible spectroscopy (HPLC-UV-Vis) [88], high-pressure size exclusion chromatography (HPSEC) [36], or ion chromatography with a refractive index detector [89]. GC is frequently used, and this analytical method requires derivatization to allow simple sugars to volatilize [90]. Although these approaches can be used to determine the quantity of each sugar in polysaccharides, less common sugars or generally charged sugar units may not be detected using a standard method and will require more specifically tailored procedures. Different methods based on nuclear magnetic resonance (NMR) systems have been developed to quantify or to profile polysaccharides. After hydrolysis, monomeric sugars and functional groups of polysaccharides can be identified and quantified [91,92,93]. Either 1H or 13C involved NMR systems are widely used to analyze polysaccharides presented in different algae species [36,59,94,95,96,97]. Moreover, 22 saccharides in raw honey, in the form of a monomer, dimer, or trimer, can be distinguished without pre-treatment using NMR based on the chemical-shift-selective filtration with total correlation spectroscopy sequence [98]. Before analysis, researchers also employed desulfation and methylation methods to determine additional structural information [99,100].

To estimate the presence of possible impurities in the extracted polysaccharides, some rapid qualitative assays for testing the existence of flavonoids, alkaloids, tannins, terpenoids, steroids, saponins, glycosides, phenols, and phlorotannins according to Evans and Trease [101] and Sofowara [102], are usually considered in characterization [59]. Those methods are all regarded as colorimetric analysis, determining the concentration of the analyte by comparing the color changes of the solution. Similarly, the total carbohydrate content and the amount of 3,6-anhydrogalactose unites can also be quantified by colorimetric methods–the phenol-sulfuric acid method [103] and the resorcinol method [104,105]. Furthermore, to measure the sulphate content, the method based on turbidity measurements using barium chloride causing the precipitation of the insoluble barium sulphate is used [106,107]. The sulphate ions originate from polysaccharide hydrolysates. As such, conductometric titration can be used to quantify charged repeating units with different pKa (acid dissociation constant) values in polysaccharides [108,109].

At the macromolecular level, the length of the polymer chains is the most significant property since it determines a variety of characteristics of polysaccharides. Although the polymer length may be defined in relation to the degree of polymerization, that is, the average number of monomer units per polymer chain, it is most typically expressed in terms of the number-averaged and weight-averaged molecular weights [65]. Because of the polymer degradation occurring in the process of extraction and purification, the weight-averaged molecular weights of polysaccharides are different. That means polysaccharides with a lower degree of polymerization exhibit higher purity [65,110]. Pozharitskaya et al. discovered that algal polysaccharides with high molecular weight exhibited promising therapeutic properties in pharmacological applications [111]. The absolute molecular weight of polysaccharides is commonly determined by methods based on light scattering techniques. Moreover, combining light scattering methods with a size-based separation method, such as gel permeation chromatography or size exclusion chromatography, yields not only the averaged molecular weights but also the molecular weight distribution and polydispersity values [112].

## 4. Hydrogel Synthesis

Hydrogel design is a vital step in developing systems with new structures and properties for multiple applications in medical, agricultural, environmental, and cosmetic fields. The hydrogel structure is determined by hydrophilic groups and the polymeric networks, basically forming three-dimensional networks through crosslinking. The presence of crosslinks prevents the dissolution of hydrophilic polymers in aqueous environments [1]. Therefore, establishing crosslinking within structures is the key factor for hydrogel preparation. Hydrogels can be crosslinked by either physical or chemical procedures [113]. The physical interaction processes include association, aggregation, crystallization, complexation, and hydrogen bonding. Chemical hydrogels are covalently crosslinked formulations prepared by chemical reactions, the addition of crosslinkers, and polymerization.

### 4.1. Hydrogel Synthesis Mechanism

**Figure 19:**
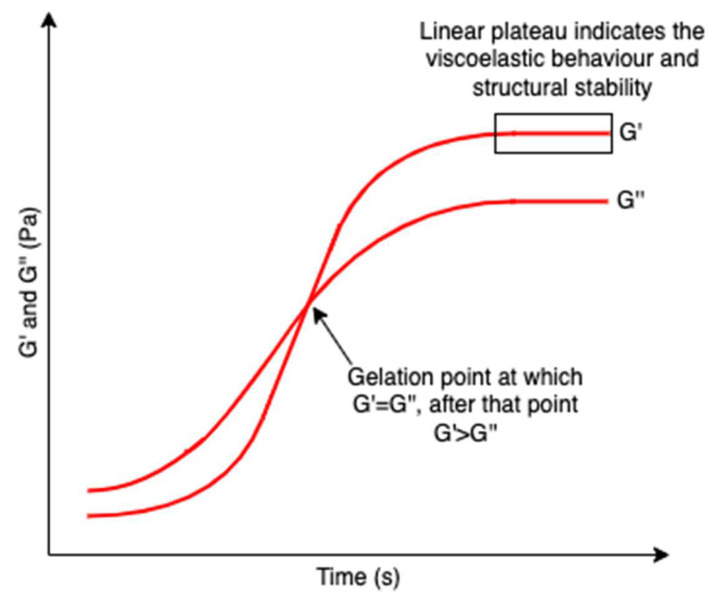
A schematic graph showing the formation of hydrogel network over time. G’ and G’’ represent the storage modulus and the loss modulus, respectively. The crossover point of G’ and G’’ indicated the gelation point, after which G’ > G’’. A linear plateau region of the storage modulus is also included revealing the viscoelastic behavior and structural stability of hydrogels.

Hydrogels are three-dimensional networks composed of hydrophilic polymer chains that are crosslinked. The hydrophilic polymeric networks result in a hydrogel with a high water swelling and absorbing capacity while remaining insoluble in water. Their test-tube inversion test (invert the test tube with the hydrogel to observe its fluidity) always shows a lack of flow due to larger storage moduli (indication of hydrogel elasticity that measures the stored deformation energy) compared with loss moduli (indication of hydrogel viscosity that measures the dissipated energy) [114] and a linear plateau region of the storage modulus which indicates the viscoelastic behavior and structural stability of a hydrogel (Figure 19) [115]; as such, they are also regarded as rheological soft solids that behave similarly to solids and have viscoelastic properties [116]. The viscoelastic properties of hydrogels correlate strongly with their microstructures, giving important information to modulate their performance [117]. These unique properties attract increasing interest as biomedical materials since hydrogels can reproduce the hydration conditions of tissues in mammals and imitate some of the physical features of the extracellular matrix based on polysaccharides (e.g., hyaluronic acids) and protein (e.g., collagen) [118,119]. Polysaccharides are representative hydrogel-forming natural polymers, which mainly originate from seaweed/algae. In recent decades, polysaccharides isolated from algae have been widely used in hydrogel production.

Algal polysaccharides-based hydrogels prepared through physical crosslinking form weak non-covalent bonding, such as van der Waals forces, electrostatic interactions, and hydrogen bonding, resulting in reversible gels based on the conformational changes. On the other hand, chemically crosslinked hydrogels develop covalent bonding between polymer molecules leading to irreversible networks based on the configurational changes [120,121]. As a result, the interactions between molecules of physically crosslinked hydrogels are easily broken with small energy, leading to the reversible sol-gel conversion [122]. The preparation of algal polysaccharides-based hydrogels, which revolves around physical and chemical crosslinking, is discussed in the following subsections. 

### 4.2. Physical Crosslinking

**Figure 20:**
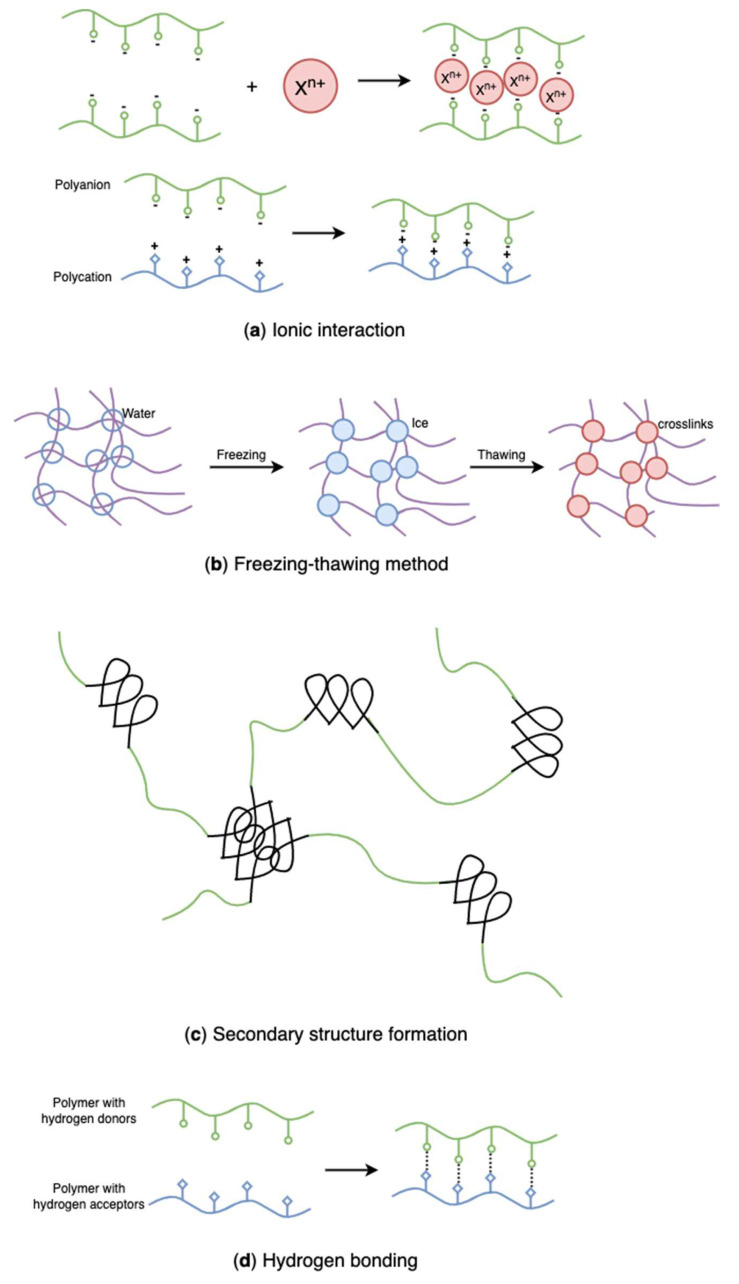
Schematic representation of physically crosslinked, algal polysaccharides-based hydrogels: (**a**) ionic interaction, (**b**) freezing-thawing method, (**c**) secondary structure formation, (**d**) hydrogen bonding.

Physically crosslinked hydrogels form three-dimensional networks by physical interactions such as hydrogen bonding, chain entanglement, van der Waals forces, and electrostatic interactions. Recently, physical crosslinking methods (Figure 20) have attracted considerable attention due to the absence of crosslinking agents in the hydrogel preparation process. These chemical crosslinkers were usually toxic and might adversely affect entrapped compounds and their bioactivity [2]. Because hydrogels are broadly used for food packaging and biomedical purposes, the research focus on hydrogel formation is gradually shifting to physical crosslink. Table 1 summarizes physical crosslinking methods commonly used for algal polysaccharides-based hydrogels. 

#### 4.2.1. Ionic Interactions

**Figure 21:**
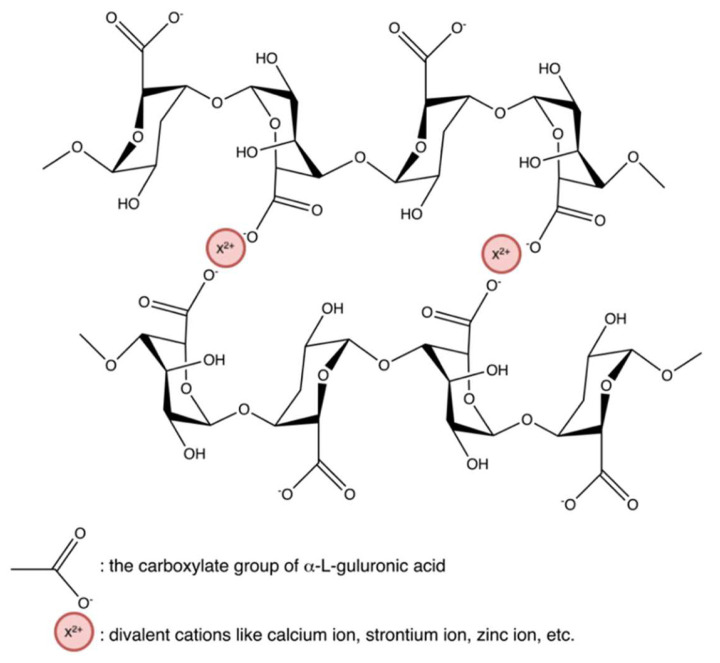
Ionic interaction between alginate and divalent actions, forming the “egg-box” structure.

Hydrogels crosslinked by ionic interaction (Figure 20a) can be achieved by adding multivalent ions with opposite charges to polyelectrolyte solutions or mixing polycations with polyanions. For example, alginates/sodium alginates are anionic biopolymers, and their common gelation process is associated with the exchange of sodium ions from alginate acids with divalent cations, such as calcium ions, strontium ions, zinc ions, and barium ions, interacting majorly with the carboxyl groups of α-L-guluronic acid to form an intermolecular crosslinking “egg-box” structure (Figure 21), in which each metal ion is coordinately bound to the carboxyl groups of guluronic acids, and the structure of guluronic acid offers the optimal distance between the carboxyl and hydroxyl groups, with a high degree of coordination with divalent cations (Table 1) [123,124,125,126]. The type of cation and corresponding concentration has been reported to impact the hydrogel properties [127]. Different cations result in required minimum concentrations, mechanical properties, and selectivity coefficient [124,127]. In comparison with magnesium ions, calcium ions exhibited stronger interactions with the alginate [124,125,128]; while compared with ions of copper, strontium, and calcium, the addition of zinc ions improved mechanical and physiological properties of the alginate-based hybrid hydrogels [129]. Furthermore, You et al., Xiao et al. and Hu et al. prepared chitosan-sodium alginate-based hydrogel via ionic gelation, in which anionic polysaccharide sodium alginate interacted with cationic polysaccharide chitosan (Table 1) [130,131,132]. The primary amino groups (−NH_2_) in the chitosan or water-soluble chitosan derivative chains serve as special joint points for both anions and small anionic molecules when they are protonated into ammonium groups (-NH_3_^+^) at a pH of 6.2 or less (pKa = 6.2) [133]. Similarly, Nuno Carvalho and his coworkers designed composite hydrogels with better mechanical properties based on the blending of marine origin biopolymers collagen, chitosan and fucoidan (Table 1) [54]. The ionic electrostatic interactions were promoted between the positively charged groups (protonated amines) of collagens and/or chitosan and the negatively charged groups (ester sulphates and carboxylates) of fucoidan. Therefore, as anionic polymers, alginate, fucoidan, and carrageenan, also usually crosslinks with various positively charged biopolymers (e.g., chitosan, starch, and cellulose) via electrostatic interaction instead of inorganic cations [53,54,130,131,132,134,135,136,137,138].

Running et al. synthesized hydrogels based on lambda-carrageenan in the presence of trivalent iron ions (Table 1) [139]. They found kappa- and iota-carrageenans showed gelation in the presence of mono- and di-valent ions, but lambda-carrageenan yielded only viscous solutions. That might be attributed to the amount of negatively charged sulphate group in every disaccharide repeating unit (Figure 4)–kappa-carrageenan has one negatively charged sulphate group showing selectivity to monovalent potassium ions, while iota-carrageenan has two sulphate groups preferring divalent calcium ions – based on that, researchers examined trivalent iron ions promoted interactions between lambada-carrageenan with three sulphate groups leading to gelation. Furthermore, because the storage moduli were much greater than the loss moduli, the iron(III)-lambda-carrageenan hydrogels were thermostable. Cao et al. identified specific binding between lambda-carrageenan and trivalent ions of iron and aluminum, but no interaction between lambda-carrageenan and trivalent chromium ions (Table 1) [26]. Mohamadnia et al. combined kappa-carrageenan and alginate to interact with divalent ions of calcium and monovalent potassium ions, forming ionically crosslinked carrageenan-alginate hydrogel beads (Table 1) [140]. Unlike the gelation mechanism of alginate-calcium cations developing an “egg-box” model, the sulphate esters and anhydro-oxygen atoms of kappa-carrageenan form an electrostatic attraction with potassium which functions as an intramolecular adhesive. Those homo-crosslinked polymer networks were held together by permanent entanglements. The resulting hydrogels exhibited a smoother surface morphology, and the carrageenan-potassium ions part appreciably enhanced the thermostability of the networks. 

It has been reported that the presence of boric acid and divalent cations in ulvan causes the formation of thermoreversible hydrogels, which involves the formation of borate-polysaccharide complexes (Table 1) [141,142,143,144]; among which divalent cations formed ionic bridges between either the carboxylic group of uronic acid and/or sulphate groups with borate or used to stabilize the coordination of borate with the hydroxyl groups of ulvan [141,142]. Lahaye et al. also indicated stronger interactions between copper cations and ulvan, while no gelation occurred in the presence of magnesium ions [145].

#### 4.2.2. Freezing-Thawing Method

While synthetic polymers have outstanding processability and mechanical properties, natural polymers are biocompatible, biodegradable, and non-toxic. Blends of natural and synthetic polymers have received much interest for biomedical applications due to their superior mechanical and thermal characteristics, as well as biocompatibility, compared with single-component materials [146,147]. Poly(vinyl alcohol) (PVA) is a non-ionic polyhydroxy polymer that is soluble in water. Due to its non-toxic, biocompatible, and gelation properties, PVA has been widely used in hydrogel formation for multiple purposes [148,149,150]. However, pure PVA hydrogels have restricted applicability owing to their poor swelling capacity and inert responsivity [151,152]. Introducing natural ionic biopolymers into PVA hydrogels and establishing in-between physical binding is an efficient method to improve the ionic behavior and the structure of PVA hydrogels. Recently, various studies have combined algal polysaccharides with PVA to develop new hydrogels with enhanced structure and properties [151,152,153,154,155,156]. As the mildest, most simple, and effective physical crosslinking method, freezing-thawing (Figure 20b) has gained considerable attention in recent years since it does not require the presence of toxic crosslinkers [151,157]. 

Such polymeric systems are designed under freezing conditions and stored in a frozen state. Subsequently, the initial solution or dispersions are thawed, establishing the precondition for gel structure formation and transition [158]. In the process, sufficient crystallization of initial systems is a necessity, so researchers usually repeat the freezing-thawing cycles. Cryogels or heterogeneous hydrogels are formed after melting down [159]. As the transformation of water to ice results in an increase in the polymer concentration, forced alignment within the chains of hydrogels builds a corresponding mechanism helping in forming side-by-side associations. After thawing out, these side-by-side associations still serve as the binding domains of the networking [27].

Jiang et al. prepared PVA/sodium alginate hydrogels with high toughness and electric conductivity by a freezing-thawing cycle (Table 1) [155]. The homogeneous solution of PVA and sodium alginate was frozen (−20 °C for 3 h) followed by being thawed (30 °C for 6 h). The process was repeated twice. Sequentially, in order to enhance strength and conductivity, the obtained hydrogel was immersed in a saturated sodium chloride solution. The operation contributed to the loss of free state water from hydrogels and the permeation of sodium and chloride ions into hydrogels. The treated PVA/sodium alginate hydrogel also resulted in the salting-out effect with increased chain entanglement. Furthermore, Hua et al. combined the ionic crosslinking and the freezing-thawing cycles (cooled at −20 °C for 48 h and then thawed at 25 °C for 4 h) to design dual-crosslinked PVA/sodium alginate hydrogels for controlled drug delivery (Table 1) [152]. The dual-crosslinked hydrogels included the pH-sensitive property of sodium alginate and the controlled drug release of PVA gels, showing improved swelling behaviors and encapsulation efficiency as well as a timed-release ability. 

Shao et al. designed a self-healing hydrogel using PVA and agarose via the freezing-thawing method (frozen at −15 °C for 8 h and then thawed at 25 °C for 12 h) and hydrogen bonding (Table 1) [154]. Firstly, agarose was converted into linear molecules at a temperature above its melting point of 85 °C during the preparation of transparent, homogenous PVA/agarose solution. After cooling down to 25 °C, PVA-containing agarose single network hydrogels were crosslinked by the double helix formed by agarose linear molecules via the hydrogen bond. The freezing-thawing cycles caused crystallized PVA to form crystallites as physical crosslinks. Agarose and PVA networks were entangled through van der Waals force and hydrogen bonding. In comparison with the pure PVA hydrogel, the hybrid hydrogel exhibited higher thermal stability and enhanced compressive strength (eight times the strength of pure PVA hydrogel). 

Mahdavinia et al. also synthesized magnetic hydrogels composed of kappa-carrageenan and PVA through the freezing-thawing method in the potassium ions-rich solution (Table 1) [156]. To crosslink the PVA component, the freezing-thawing process, in which the obtained solution containing PVA, kappa-carrageenan, and iron(II,III) oxide was frozen overnight and thawed at ambient temperature for 5 h, was used. After four freezing-thawing cycles, the hydrogels were immersed in the potassium chloride to crosslink the kappa-carrageenan component. To achieve the removal of cationic substances, researchers innovatively incorporated iron salts in the binary hydrogels to obtain a magnetic property. Although the introduction of magnetic nanoparticles into hydrogels caused a decrease in dye adsorption, increasing the ratio of kappa-carrageenan in the hydrogel composition could improve the adsorption ability of hydrogels. 

#### 4.2.3. Secondary Structure

The formation of a secondary structure (Figure 20c) refers to a three-dimensional network polymer adopted by the macromolecular chains. The formation process may involve a coil-to-helix transition. Once constructed, the helix can aggregate to establish points of contact between the polymer chains, resulting in creating a three-dimensional network. Electrostatic interpolymer chain repulsions induce the formation of helical aggregation, and then the structure is stabilized by weak attractive interactions. In these algal polysaccharide-based hydrogel systems, the helices may be broken owing to twists caused by the irregularity in the polymer chains, which consequently regulates the size of the crosslinking points [65].

Forget et al. reported hydrogels produced from algal polysaccharides, such as agarose and kappa-carrageenan possessing an α-helical backbone, could be customized by inducing a changeover in the secondary structure from an α-helix (a right hand-helix conformation) to a β-sheet (a generally twisted, pleated sheet) by carboxylation (Table 1) [28]. They blended α-helix-rich native agarose/kappa-carrageenan with the β-sheet-rich carboxylated derivative in the hot deionized water, achieving the conversion of an α-helix to a β-sheet; followed by an aggregation of polymer chains through β-sheet motifs and elongation of these aggregates into a high-aspect-ratio structure. The secondary structure formation enabled the gel modulus to be tuned over four orders of magnitude. Physicochemical properties of hydrogels could be tunable by switching their secondary structure from an α-helix to a β-sheet. The resulting hydrogels were examined to possess tailored mechanical properties as well as predictable roughness, fibre organization, and shear modulus [160]. Moreover, to further control the gel characteristics, the polysaccharide content can be increased to promote helix aggregation, which results in a higher gel strength [161,162]. 

When cations are present, both lambda- and kappa-carrageenan undergo a coil-to-helix transition, resulting in the formation of double helices. The development of a helix is followed by subsequent helix aggregation in kappa-carrageenan [163,164,165]. Still, this aggregation does not occur in lambda-carrageenan owing to the presence of two sulphate groups, which induces a greater electrostatic chain repulsion [166,167]. The gelation of kappa-carrageenan relies on monovalent cations [168]. The kind of cation used to initiate the gelation process influences the mechanical characteristics of hydrogels. For example, kappa-carrageenan forms a stronger gel when combined with potassium ions [168,169,170,171]. However, some anions, such as iodide anions, have been designed to attach to the helix, which affects the gelation mechanism, hindering helix aggregation and gelation [172,173,174]. Because the development of lambda- and kappa-carrageenan hydrogels is driven by their secondary structure, manipulating this structure, such as adding ions, may have a remarkable effect [65]. Additionally, single helices created by a single carrageenan chain [169,175] are different from double helices formed by the interweaving of two carrageenan chains [163,176] or the intramolecular cycling or hair pinning of a single carrageenan chain [177,178]. Voron’ko et al. prepared kappa-carrageenan/gelatin-based hydrogels with helical structures (Table 1) [179]. The process consisted of a mixture of anionic kappa-carrageenan and gelatin, the formation of self-assembling polyelectrolyte complexes, a coil to helix transition of gelatin, and subsequent aggregation of helices. The gelatin and kappa-carrageenan helices supported the stabilization of hydrogel networks. Kappa-carrageenan serving as host-polyelectrolyte, interacted with guest-polyelectrolyte gelatin, forming complex hydrogels in the presence of hydrophobic interaction, electrostatic interaction, hydrogen bond, and the secondary structure of triple collagen-like helices of gelatin and intramolecular double helices of kappa-carrageenan. By increasing the kappa-carrageenan/gelatin ratio, the gelation ratio would slow down.

#### 4.2.4. Hydrogen Bonding

Hydrogels can also be formed through other secondary bonding–hydrogen bonding (Figure 20d)–because of the functional groups in polymers. Researchers have successfully prepared hydrogen-bonded hydrogels using polymers with carboxyl groups along their chains by lowering the pH of their aqueous solutions. In the research of Jing et al., novel sodium alginate/carboxymethyl chitosan hydrogel beads promoted by hydrogen bond were designed (Table 1) [25]. The hydrogel beads were prepared by dropping the blends of two polymers into the citric acid solution and showed excellent pH sensitivity and protein encapsulation capacity. Likewise, a hydrogen-bonded carboxymethyl cellulose-based hydrogel was prepared by dispersing carboxymethyl cellulose into hydrogen chloride and citric acid, in which the sodium in the carboxyl group was replaced with hydrogen in acidic solutions leading to the aggregation of carboxymethyl cellulose molecules (Table 1) [180]. However, the conversion of all the −COONa into −COOH in carboxymethyl cellulose results in excess crosslinks giving unstable and brittle hydrogels. That indicates that some carboxyl groups remaining in the form of -COONa help in stabilizing hydrogels. Decreasing acid concentration and increasing temperature also favour the stability of hydrogels. Additionally, Wang et al. prepared a fucoidan and kappa-carrageenan blend hydrogel through hydrogen bonding (Table 1) [181]. The addition of kappa-carrageenan promotes non-gelling fucoidan gelation, forming a strong entanglement network through hydrogen bonds, and the rheological properties, thermal stability, water retention, and frost resistance of fucoidan were improved by kappa-carrageenan. 

**Table 1:** Physical crosslinking methods for algal polysaccharides-based hydrogel preparation.

Crosslinking	Materials	Important reagents	Gelation	Properties	Ref.
Ionic interaction	Alginate/alginate sodium (brown algae such as *Laminaria hyperborea* and *lessonia*)	Divalent cations-containing solutions, such as calcium chloride, zinc chloride, etc.	Divalent cations interacted with their carboxyl groups to form intermolecular crosslinking “egg-box” structures	Biocompatible, biodegradable, divalent cations-affected mechanical properties	[124,125,126,128,129,130]
	Alginate/Sodium alginate (brown algae) Chitosan derivatives	5% acetic acid or other dilute organic/inorganic acids	Anionic sodium alginate interacted with cationic chitosan derivatives	pH-sensitive, biocompatible, biodegradable, high capacity to bind heavy metal ions, acidic gas, and basic gas	[130,131,132]
	Fucoidan (brown algae *Fucus vesiculosus*) Collagen Chitosan	-	Ionic electrostatic interactions between the positively charged groups of collagens and/or chitosan and the negatively charged groups of fucoidan	Degradable, biocompatible	[54]
	Lambda-carrageenan (red algae *Sarcothalia lanceata*)	Aluminium(III) chloride/iron(III) chloride/iron(III) chloride hexahydrate	Ionic interactions in the presence of specific trivalent ions	Thermostable, biocompatible, biodegradable	[26,139]
	Kappa-carrageenan (red algae) Sodium alginate (brown algae)	Calcium chloride and potassium chloride	Combined algal polysaccharides to interact with divalent calcium ions and monovalent potassium ions, forming alginate-calcium cation and kappa-carrageenan-potassium cation crosslinked networks	Thermostable, biocompatible, biodegradable	[140]
	Ulvan (green algae *Ulva* spp.)	Borate, calcium chloride	Boric acid and divalent cations such as calcium cations initiated ionic crosslinking. It also involved the chelation of calcium with hydroxyl groups of borates	Thermoreversible, biocompatible, biodegradable	[142]
Freezing-thawing method	Sodium alginate (brown algae) PVA	Calcium chloride, and/or diclofenac sodium (changed from transparent to white and opaque solution)	Repeated freezing-thawing cycles on PVA-containing ionically crosslinked sodium alginate hydrogels for two times	Biocompatible, pH-sensitive, improved swelling behaviors and encapsulation efficiency	[152]
Freezing-thawing method	Agarose (red algae Rhodophyta) PVA	-	Thermal-induced aggregation (above 85 °C) of agarose followed by fabricating PVA hydrogels via the repeating freezing-thawing cycles. They entangled through van der Waals force and hydrogen bonding	Robust mechanical property, biocompatible, self-healing	[154]
	Sodium alginate (brown algae) Poly(vinyl alcohol) (PVA)	Sodium chloride	Repeated freezing-thawing cycles on the homogeneous PVA/sodium alginate solution and then immersed the virgin hydrogel in the saturated sodium chloride solution	Biocompatible, high toughness and electric conductivity	[155]
	Kappa-carrageenan (red algae) PVA	Iron salts (iron(II) sulfate heptahydrate and iron(III) chloride hexahydrate), ammonia solution, potassium chloride	Mixed iron salts, PVA and kappa-carrageenan, followed by adding ammonia solution to adjust pH at 10 until magnetic nanoparticles with the dark color were formed. The polymer networks were crosslinked by the repeating freezing-thawing cycles and then ionic interaction with potassium cations	Magnetic	[156]
Secondary structure	Native agarose (red algae *Gelidium* and *Gracilaria*) Kappa-carrageenan (red algae)	(2,2,6,6-Tetramethylpiperidin-1-yl)oxyl (TEMPO), sodium bromide, sodium hypochlorite, sodium hydroxide, ethanol, sodium chloride, and sodium borohydride	Blended α-helix-rich agarose/kappa-carrageenan with the β-sheet-rich carboxylated derivatives in the hot deionized water, achieving converting an α-helix to a β-sheet. Followed by aggregation of polymer chains through β-sheet motifs and elongation of these aggregates into high-aspect-ratio structure	Injectable, tunable mechanical and structural properties, biocompatible, biodegradable, formation in vivo	[28,161]
	Kappa-carrageenan (red algae) Gelatin	-	Host (kappa-carrageenan)-guest (geletin) interaction generating electrostatic interaction, hydrophobic interaction, and hydrogen bonding, a coil to helix transition of gelatin, followed by aggregation of helices	Biocompatible, biodegradable	[179]
Hydrogen bonding	Sodium alginate (brown algae) Carboxymethyl chitosan	Citric acid	Blended those two biopolymers in the citric acid solution, resulting in hydrogen bonding between the polymers and citric acid under an acidic environment	pH-sensitive, thermally stable, biocompatible, biodegradable	[25]
	Sodium carboxymethyl cellulose (not specified)	Hydrochloric acid/citric acid	Mixed sodium carboxymethyl cellulose with acid, replacing sodium in carboxymethyl group with hydrogen. Carboxymethyl cellulose molecules aggregated because of reduction of the polymer solubility in water	Stable, biocompatible, durable	[180]
Hydrogen bonding	Fucoidan (brown algae) Kappa-carrageenan (red algae)	-	Non-gelling polysaccharide fucoidan interacted with kappa-carrageenan under high temperature (approximately 95 °C), forming hydrogel bonds	Biocompatible, biodegradable, improved water retention and frost resistance, thermal stable, enhanced rheological properties	[181]
Freezing-thawing method	Agarose (red algae *Rhodophyta*) PVA	-	Thermal-induced aggregation (above 85 °C) of agarose followed by fabricating PVA hydrogels via the repeating freezing-thawing cycles. They entangled through van der Waals force and hydrogen bonding	Robust mechanical property, biocompatible, self-healing	[154]
	Sodium alginate (brown algae) Poly(vinyl alcohol) (PVA)	Sodium chloride	Repeated freezing-thawing cycles on the homogeneous PVA/sodium alginate solution and then immersed the virgin hydrogel in the saturated sodium chloride solution	Biocompatible, high toughness and electric conductivity	[155]
	Kappa-carrageenan (red algae) PVA	Iron salts (iron(II) sulfate heptahydrate and iron(III) chloride hexahydrate), ammonia solution, potassium chloride	Mixed iron salts, PVA and kappa-carrageenan, followed by adding ammonia solution to adjust pH at 10 until magnetic nanoparticles with the dark color were formed. The polymer networks were crosslinked by the repeating freezing-thawing cycles and then ionic interaction with potassium cations	Magnetic	[156]
Secondary structure	Native agarose (red algae *Gelidium* and *Gracilaria*) Kappa-carrageenan (red algae)	(2,2,6,6-Tetramethylpiperidin-1-yl)oxyl (TEMPO), sodium bromide, sodium hypochlorite, sodium hydroxide, ethanol, sodium chloride, and sodium borohydride	Blended α-helix-rich agarose/kappa-carrageenan with the β-sheet-rich carboxylated derivatives in the hot deionized water, achieving converting an α-helix to a β-sheet. Followed by aggregation of polymer chains through β-sheet motifs and elongation of these aggregates into high-aspect-ratio structure	Injectable, tunable mechanical and structural properties, biocompatible, biodegradable, formation in vivo	[28,161]
	Kappa-carrageenan (red algae) Gelatin	-	Host (kappa-carrageenan)-guest (geletin) interaction generating electrostatic interaction, hydrophobic interaction, and hydrogen bonding, a coil to helix transition of gelatin, followed by aggregation of helices	Biocompatible, biodegradable	[179]
Hydrogen bonding	Sodium alginate (brown algae) Carboxymethyl chitosan	Citric acid	Blended those two biopolymers in the citric acid solution, resulting in hydrogen bonding between the polymers and citric acid under an acidic environment	pH-sensitive, thermally stable, biocompatible, biodegradable	[25]
	Sodium carboxymethyl cellulose (not specified)	Hydrochloric acid/citric acid	Mixed sodium carboxymethyl cellulose with acid, replacing sodium in carboxymethyl group with hydrogen. Carboxymethyl cellulose molecules aggregated because of reduction of the polymer solubility in water	Stable, biocompatible, durable	[180]
Hydrogen bonding	Fucoidan (brown algae) Kappa-carrageenan (red algae)	-	Non-gelling polysaccharide fucoidan interacted with kappa-carrageenan under high temperature (approximately 95 °C), forming hydrogel bonds	Biocompatible, biodegradable, improved water retention and frost resistance, thermal stable, enhanced rheological properties	[181]

### 4.3. Chemical Crosslinking

**Figure 22:**
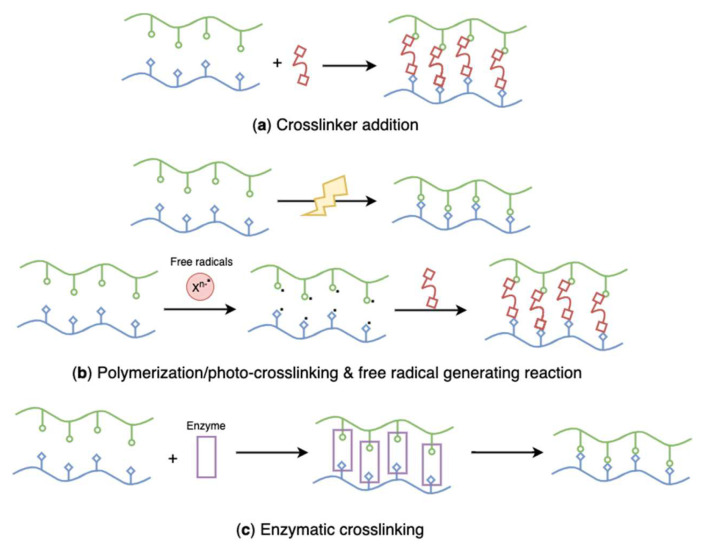
Schematic representation of chemically crosslinked, algal polysaccharides-based hydrogels: (**a**) crosslinker addition, (**b**) polymerization/photo-crosslinking and free radical generating reaction, (**c**) enzymatic crosslinking.

Physically crosslinked hydrogels based on algal polysaccharides are attractive materials with broad applications due to their biocompatibility, biodegradability, non-toxicity, and the absence of poisonous crosslinkers. However, they are susceptible to external stimuli because of the reversible nature of interactions between polymer chains, and the gel to sol transformation degrades the designed structure. Thus, some chemically crosslinking methods (Figure 22) have been introduced to prepare polysaccharide-based hydrogels with controllable properties and irreversible networks. Table 2 presents some typical chemically crosslinked, algal polysaccharides-based hydrogels.

#### 4.3.1. Crosslinker Addition

Crosslinker addition (Figure 22a) is the most important strategy that has been extensively used to prepare chemically crosslinked hydrogels. Multifunctional polymers and photosensitive or enzyme-catalyzed agents are commonly employed as chemical crosslinkers [182]. Zhang et al. developed composite agarose/hyaluronic acid hydrogels with epichlorohydrin (a bifunctional alkylating agent [183]) as a crosslinking agent (Table 2) [184]. The study found that the hybrid hydrogels showed no cytotoxic effect, although epichlorohydrin is classified as a probable human carcinogen. 

However, most crosslinkers possess toxicity and unknown biocompatibilities. To prevent the presence of crosslinkers and reaction by-products, multiple purifications are required before administration. On the other hand, crosslinkers may react with loaded active pharmaceutical ingredients, affecting the therapeutic effects [2]. As such, the main drawback of this method is the limited choice of safe and biocompatible crosslinking agents. Genipin is a representatively biocompatible crosslinker extracted from the *gardenia* (a flowering plant) [185]. It has been reported to be effective in crosslinking polymers with amino groups and demonstrates minor cytotoxicity compared with conventional chemical crosslinkers, such as glutaraldehyde [186]. Li et al. fabricated genipin crosslinked composite hydrogels comprised of kappa-carrageenan and chitosan (Table 2) [187]. Remarkably, pristine chitosan hydrogels prepared by the phase inversion technique were further fabricated with the genipin crosslinker, leading to hydrogels with improved mechanical properties under weak basic conditions. This novel hydrogel improved anticoagulant and antibacterial properties compared with the chitosan-based hydrogel. Similarly, Chen et al. also designed alginate-chitosan hydrogels composed of alginate cores and genipin crosslinked chitosan membrane, possessing effective resistance to mechanical force, calcium sequestration, enzyme degradation, and gastrointestinal impediments (Table 2) [188,189]. 

Additionally, polymers and hydrogels can be further reinforced by adding crosslinkers. Zhao et al. prepared cellulose (cotton linter pulp) hydrogels using sequential chemical and physical crosslinking, in which epichlorohydrin was introduced as the crosslinker, followed by hydron bonding and chain entanglement [190]. The incorporation of chemically crosslinked and physically crosslinked domains afforded cellulose-based hydrogels with relatively high stiffness, high toughness, and good recoverability compared to the single crosslinked hydrogel. Campos et al. chemically synthesized membrane-like hydrogels based on agarose and fibrin with genipin (Table 2) [19]. Researchers fabricated fibrin-agarose hydrogels using ionic interactions, followed by adding the crosslinking agent genipin to improve the structural and biomechanical properties. 

Porphyrins have been proven their loading efficiency is limited [191,192], and they cannot form supramolecular gels by molecular self-assembly (intermolecular interactions that are established between molecules or structural motifs [193]). As a result, they have been migrated out of hydrogel materials. However, they can be synthesized as crosslinking agents [194]. Lovell et al. prepared porphyrin crosslinked hydrogels using a rapid stepwise condensation copolymerization reaction between poly(ethylene glycol) (PEG) and porphyrin (Table 2) [191]. The designed hydrogels were examined to have stable, alternating porphyrin and PEG subunits that provided significant near-infrared optical properties, allowing long-term, non-invasive fluorescence monitoring and image-guided surgical resection in vivo. So, the proposed hydrogels are ideal biomedical materials for fluorescence-guided monitoring and surgical resection.

#### 4.3.2. Polymerization

Except for reactions with crosslinkers, polymerization (Figure 22b) through free radical crosslinking is also the most common method for chemical hydrogel preparation. Free radical polymerization, also known as photo-crosslinking, is a chemical crosslinking process involving free radical initiators, such as methacrylate, benzoyl peroxide, and ammonium peroxodisulphate [195]. To make polymers photopolymerizable, they are modified with specific unsaturated functional groups (e.g., acrylates) that undergo free-radical polymerization in the presence of a photoinitiator when exposed to ultraviolet (UV) light. Custódio et al. designed photo-crosslinked laminarin-based hydrogels to develop a new injectable biomedical system using UV irradiation (Table 2) [20]. Laminarin was not photo-crosslinkable, which requires chemical modification with acrylate groups before the hydrogel preparation. In the reaction process, 2-hydroxy-4′-(2-hydroxyethoxy)-2-methylpropiophenone was used as a photoinitiator. The rapid gelation triggered by the low dose UV irradiation helped in the encapsulation of bioactive ingredients and cells, avoiding bioactive damage. Similarly, Feng et al. modified hydroxyl groups of laminarin with glycidyl methacrylate, followed by the photopolymerization of methacrylated laminarin via a radical reaction under UV irradiation [196]. Morelli et al. synthesized ulvan-based hybrid nanogels through radical copolymerization of N-vinylcaprolactam monomer onto suitably modified ulvan with acrylate groups (Table 2) [17]. The radical copolymerization process was induced by UV irradiation. In the preparation process, modified ulvan acted as not only the grafting from macromer during the polymerization of N-vinylcaprolactam but also a crosslinker during the synthesis of thermoresponsive Poly(N-vinylcaprolactam) networks. Apart from attaching photosensitive functional groups to polymers, researchers also combined algae-extracted polysaccharides with photo-crosslinkable polymers to synthesize composite hydrogels. El-Din et al. synthesized PVA/sodium alginate hydrogels by electron beam irradiation (Table 2) [22]. In this study, researchers found that the content of non-photo-crosslinkable sodium alginate could not exceed 40%; otherwise, no homogenous hydrogel could be produced.

Hydrogelation can also be induced by other high-energy radiation such as gamma rays [21,197] or electron beams [22,198]. High energy radiation has been proven to be a powerful and fast tool to sterilize and tailor the properties of algal polysaccharides-based hydrogels. It can also be used to treat prepared polysaccharides hydrogels to obtain customized mechanical, structural, and chemical properties for desired applications. For instance, Singh and her coworkers synthesized PVP-alginate hydrogels by gamma irradiation; meanwhile, they also included silver dressings into the prepared hydrogel using gamma rays, designing a tailored composite hydrogel with the antimicrobial property (Table 2) [21]. Notably, due to the different dissolution characteristics of alginate and PVP, the hydrogel content decreased as the amount of alginate increased. Krömmelbein et al. treated agarose hydrogels under 10 MeV electron beam [199]. The high energy electron irradiation tailored their properties as well as rheology and average molecular weight of agarose. Compared with UV- and gamma-rays, electron beam radiation is extremely effective owing to its large penetration depths and high dosage rates [197,199]. 

Moreover, polymerization can also be initiated by a free radical-generating system, which is regarded as a vinyl addition polymerization reaction (Figure 22b). Duman et al. prepared agarose/kappa-carrageenan composite hydrogel through a free radical crosslinking reaction in the presence of ammonium persulfate as a radical initiator, tri(ethylene glycol) divinyl ether (TEGDE) as a crosslinker, and N,N,N′,N′-tetramethylethylenediamine (TEMED) as a catalyst (Table 2) [11,200]. TEMED accelerates the formation rate of free radicals from persulfate, which catalyzes polymerization. The addition of ammonium persulfate generated negatively charged sulphate radicals, and then those radicals replaced hydrogens in the hydroxyl groups of the polysaccharides forming alkoxy radicals to crosslink with the crosslinker TEGDE.

#### 4.3.3. Enzyme-Catalyzed Reaction

Crosslinking using enzymes (Figure 22c) is a novel approach used to form regular hydrogels or in situ hydrogels under mild conditions, such as physiological environments [2]. 

Enzymatically crosslinked ulvan-based hydrogels were prepared by blending ulvan with tyramine under the catalyzation of horseradish peroxidase enzyme, in which hydrogen peroxide was used as a reagent (Table 2) [23]. The tyramine modification caused ulvan to be susceptible to enzymatic recognition; as such, the polysaccharides could be crosslinked through oxidative coupling. Likewise, Hou et al. designed enzymatically crosslinked dopamine- or tyramine-alginate conjugates via the oxidative coupling of phenol or aniline moieties in the presence of horseradish peroxidase enzyme and hydrogen peroxide (Table 2) [24]. This method has emerged as an important method to synthesize in situ injectable hydrogels. Compared with photopolymerization, the enzyme-catalyzed reaction for in situ gelation shows its superiority – no photosensitizers and prolonged irradiation required – so that local temperature does not increase drastically, leading to damage to surrounding cells tissues [201]. However, it is necessary to reduce the concentration of hydrogen peroxide in future improvements to avoid its cytotoxicity [24].

**Table 2:** Chemical crosslinking methods for algal polysaccharides-based hydrogel preparation.

Crosslinking	Materials	Important reagents	Gelation	Properties	Ref.
Crosslinker addition	Agarose (red algae) Hyaluronic acid	Epichlorohydrin as a crosslinker, sodium hydroxide	Mixed alkali-treated agarose and hyaluronic acid together and reacted with chemical crosslinker epichlorohydrin	Non-cytotoxic, biodegradable, biocompatible, thermal stable	[184]
	Kappa-carrageenan (red algae) Chitosan	Genipin as a crosslinker, sodium hydroxide, potassium chloride	Pristine physically-crosslinked chitosan hydrogels (phase inversion technique) reacted with crosslinking agent genipin, developing inner chitosan core and then immersed in carrageenan solution forming outer carrageenan shell in the presence of potassium ions	Biocompatible, anticoagulant, antibacterial	[187]
	Fibrin Agarose (red algae)	Calcium chloride, genipin as a crosslinker	Ionically crosslinked fibrin-agarose hydrogels were subject to chemical crosslinking with genipin	Biocompatible, biodegradable, biomimetic, enhanced structural and biomechanical properties	[19]
	Sodium alginate (brown algae) Chitosan	Calcium chloride, genipin as a crosslinker	Ionically crosslinked alginate hydrogels immersed into chitosan forming alginate-chitosan microcapsules. The microcapsules were further crosslinked by genipin	Biocompatible, stable, strong resistance to mechanical shear forces, calcium sequestration, gastrointestinal impediments, and enzymatic degradation	[188,189]
	Poly(ethylene glycol) (PEG)	Porphyrin (meso-tetrakis(4-carboxyphenyl) porphine) as a crosslinker	Synthesized porphyrin as a crosslinker to crosslink PEG through a condensation copolymerization reaction	Near-infrared optical properties, stable	[191]
UV-initiated polymerization	Laminarin (brown algae *Laminaria* and *Eisenia*)	Glycidyl methacrylate, dimethyl sulfoxide, 4-(N,N-dimethylamino)pyridine as a catalyst in the methacrylation, 2-hydroxy-4′-(2-hydroxyethoxy)-2-methylpropiophenone as a photoinitiator	Methacrylated laminarin was subjected to UV irradiation (320–500 nm) at 5–8 mW/cm^2^ for seconds	Injectable, biocompatible, mechanically stable, low viscosity	[20]
UV-initiated polymerization	Ulvan (green algae *Ulva armoricana*) N-vinylcaprolactam	Acryloyl chloride, sodium hydroxide, 2-hydroxy-4′-(2-hydroxyethoxy)-2-methylpropiophenone as a photoinitiator	The synthesis involved the grafting copolymerization of N-vinylcaprolactam onto the side chains of acryloyl chloride-modified ulvan through UV irradiation (400 W, 365 nm, 8–10 mW/cm2, 70 °C) promoted radical process	Thermoresponsive, biocompatible, increased loading efficiency	[17]
Gamma rays-initiated polymerization	Alginate (brown algae) Poly(vinyl pyrrolidone) (PVP)	Silver nitrate	Combination of PVP and alginate was gamma irradiated at different doses of 25 and 40 kGy at a dose rate of 5.54 kGy/h. Nanosilver was incorporated in the PVP-alginate hydrogel using gamma radiation at 25 kGy, forming a composite hydrogel	Efficient fluid absorption capacity, biocompatible	[21]
Electron beam-initiated polymerization	Sodium alginate (brown algae) PVA	-	Exposed the completely miscible sodium alginate and PVA solutions under electron beam irradiation at a constant dose of 25 kGy of accelerated electrons	Highly hydrophilic, thermal stable, pH-responsive, temperature responsive, biocompatible	[22]
Free radical generating reaction (vinyl addition polymerization)	Agarose (red algae *Rhodophyceae*) Kappa-carrageenan (red algae)	Ammonium persulfate and N,N,N′,N′-tetramethyl ethylenediamine (catalyst) as a radical initiator-accelerator pair, tri(ethylene glycol) divinyl ether as a crosslinker	The addition of ammonium persulfate generated negatively charged sulphate radicals. The sulfate anion radical replaced hydrogen in the hydroxyl group of the polysaccharide substrate, forming alkoxy radicals. Crosslinking happened between alkoxy radicals and crosslinkers	Improved adsorption capacity, non-Fickian swelling, biocompatible	[11,201]
Enzyme-catalyzed reaction	Ulvan (green algae *Ulva armoricana*)	Tyramine hydrochloride, N-(3Dimethylaminopropyl)-N′-ethylcarbodiimide hydrochloride, N-hydroxysulfosuccinimide sodium salt, hydrogen peroxide, and horseradish peroxidase enzyme	Tyramine-modified ulvan was sensitive to horseradish peroxidase enzyme and then crosslinked polysaccharides through oxidative coupling	Injectable, biocompatible	[23]
	Alginate (giant brown seaweed)	Dopamine hydrochloride or tyramine hydrochloride, 1-ethyl-3-(3-dimethylaminopropyl) carbodiimide hydrochloride, N-hydroxysulfosuccinimide, hydrogen peroxide, and horseradish peroxidase enzyme	Crosslinked dopamine- or tyramine-modified alginate via the oxidative coupling of phenol or aniline moieties in the presence of horseradish peroxidase enzyme and hydrogen peroxide	Improved adhesion, in situ gelling, biocompatible	[24]

### 4.4. Semi-Interpenetrating and Interpenetrating Networks

**Figure 23:**
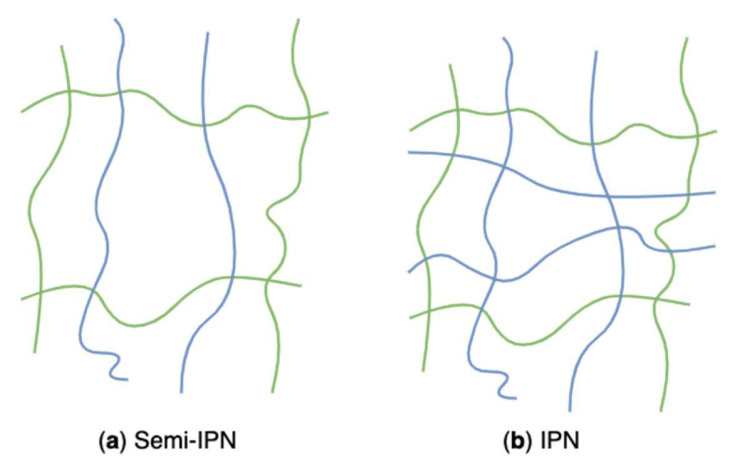
Schematic representation of algal polysaccharides-based semi-IPN (**a**) and IPN (**b**) networks.

Algal polysaccharides-based hydrogels can also be classified as two composite types: semi-interpenetrating networks (semi-IPN) (Figure 23a) and interpenetrating networks (IPN) (Figure 23b) according to polymeric composition and preparation methods. Those hybrid hydrogels have emerged as innovative materials for biomedical applications. Those structures combining the favourable properties of each polymeric component of the semi-IPNs or IPNs have attracted considerable interest. Their potentials lead to new systems with properties that are superior to those consisting of single components [202]. A linear polymer, such as alginate, agarose, and carrageenans, physically gets through another crosslinked network, but they do not covalently bond with each other. That is called a semi-IPN [203]. The grafted linear polymer modifies pore size and improves the slow release of loaded substances while simultaneously helping maintain the rapid kinetic response to external stimuli because of the absence of a restricting interpenetrating elastic network [120]. For example, semi-IPN hydrogels based on sodium alginate grafted polyacrylamide exhibited pH sensitivity, enhanced pH reversibility, and improved drug entrapment capacity, which was associated with the sodium alginate content [204]. Researchers conducted an in-situ free radical copolymerization between acrylamide and a bifunctional monomer in the aqueous solution of sodium alginate. During the process, semi-IPN hydrogels were formed through growing radicals of monomers and sodium alginate macroradicals. Algal polysaccharides-based hydrogels prepared from the freezing-thawing method (grafting algal polysaccharides to PVA hydrogel) are also regarded as semi-IPN networks. 

The interpenetrating network can be obtained through inextricably combining two polymers, and at least one of them is synthesized or crosslinked when the other is present [205]. These polymers are partially interweaved on a molecular scale, but no covalent bond is formed within them. Inter penetrating networks are prepared by soaking a pre-polymerized hydrogel into a monomer solution and a polymerization initiator [120]. They possess dense hydrogel matrices resulting in more robust and controllable mechanical and physical properties and a higher drug loading capacity [206]. In drug delivery systems, the pore sizes and surface chemical compositions of the interpenetrating network are tunable so that the drug release kinetics, mechanical properties, and the interaction between hydrogels and the neighbouring tissues can be adjusted [207]. Kulkarni et al. synthesized a pH-responsive IPN hydrogel based on polyacrylamide grafted kappa-carrageenan and sodium alginate through free radical polymerization, followed by the addition of ionic cations and crosslinkers [208]. The prepared hydrogels showed pulsatile swelling and its drug release behavior in response to pH.

## 5. Performance of Seaweed Polysaccharide-Based Hydrogels

To design polysaccharide-based hydrogels with the desired performance and structure that can be well fitted to a certain application, the characterization of the hydrogel network is of great significance. In general, the characterization methods consist of structural analysis and functional analysis. Additionally, the assessment of cytotoxicity and biocompatibility is indispensable to demonstrate the safety and efficacy of hydrogels prepared for biomedical applications.

### 5.1. Morphology

Morphological and structural characterization is one of the most critical analytical methods that helps identify the hydrogel morphologies–the porous structure–the porosity affects the water absorption capacity and swelling kinetics of hydrogels [209]. Through a specific analytical technique–scanning electron microscopy (SEM), atomic force microscopy (AFM), or laser scanning confocal microscopy (LSCM)–researchers validate whether the tested hydrogels possess porous structure, as well as whether those pores allow water penetration and represent interaction sites for external stimuli and hydrophilic groups of hydrogels. Those techniques are conducive to capturing the surface morphology and topography of hydrogels and offer advice on their applications, such as their effectiveness as drug delivery systems. Moreover, by exploring the porous structures, the level of swelling can be observed [209,210]. 

SEM is the most popular technique for detecting the porous structure (formation, size, and shape) and crosslinks of hydrogels, as well as the effect of loading substances [211,212,213]. Before SEM analysis, hydrogels to be tested are treated with formalin and then freeze-dried under −52 °C for six hours, subsequently cutting from the cross-sections and sputtering with gold or gold/platinum under a vacuum [210,214]. Later, Shen et al. skipped the pre-treatment and dehydration instead of using a liquid nitrogen snap freezing [215]. However, some research claimed the liquid nitrogen snap freezing caused the shrinking of gel structures, eventually resulting in an inaccurate evaluation [216,217]. Although SEM is a powerful tool that is capable of acquiring hydrogel structure information, it still has limitations; for example, it only generates two-dimensional images, and its dehydration pre-treatment results in structural collapse. As a result, AFM and LSCM usually serve as alternatives providing supplemental information [113,209]. Rahman et al. reviewed the morphological characterization of hydrogels prepared directly from cellulose or its derivatives using different instruments [209]. The review article highlighted that researchers mainly used SEM to explore the porosity and nature of the structure of cellulose-based hydrogels, while simultaneously they applied AFM to evaluate topological characteristics of hydrogels to exhibit the uniformity of surface roughness of such hydrogels. AFM can also reveal the mechanical properties of the polymer structures [218]. Compared with SEM, no special pre-treatment is required prior to AFM analysis; however, AFM is a time-consuming measuring process with relatively small measured sample areas [65]. Furthermore, researchers use LSCM to analyze the oriented structure within multilayered hydrogels [209] and use magnetic resonance imaging and X-ray tomography to measure the pore size of hydrogels in the wet state [219,220]. Although a series of techniques are available for analysis purposes, contrast and resolution play significant roles in imaging, affecting analysis results [221].

### 5.2. Swelling and Diffusion, Sensitivity, Texture and Strength, Rheology, and Transparency

Hydrogel water content varies as its surrounding environment changes. The quantity of water per unit mass of the dry sample, which is defined as the swelling degree/swelling ratio (1), is, therefore, a crucial metric for the evaluation of swelling behavior [222,223,224,225,226]. The concept of hydrogel swelling is also important for controlled release systems applied in the biomedical, agricultural, and cosmetic sectors. Because of the presence of hydrophilic groups and the electrostatic repulsions of the amphiprotic groups in the polymeric chains, the solvent molecules are absorbed into the three-dimensional network in the swelling process, resulting in the extrusion of polymeric chains and a thermodynamic swelling force of the polymeric chains being balanced [12,227]. The change of water absorbed impacts the mechanical properties of hydrogels; as such, samples should always be tested in a swollen and equilibrated condition to provide a representative value of their performance during the actual application [228]. The swelling behavior is associated with the number of crosslinking junction points–swelling is reduced if there are more crosslinking connections–so a proper crosslinking control is necessary for the hydrogel fabrication [229].

(1) $$\mathrm{Swelling}\;\mathrm{degree}\;=\;\mathrm{swelling}\;\mathrm{ratio}\;=\frac{m_{swollen}-m_{dry}}{m_{dry}}$$

where $m_{swollen}$ and $m_{dry}$ represent the weight of swollen and dried hydrogels, respectively.

On the other hand, the diffusion of solvents into three-dimensional networks is expressed using the power law Equation (2) [230,231].

(2) $$\frac{M_{t}}{M_{equilibrium}}=kt^{n}$$

Where $M_{t}$ and $M_{equilibrium}$ represent the weight of hydrogels at a specific infiltration time *t* and at an equilibrium state, respectively; and the coefficient *k* is proportional to the spread of solvents into the networks. 

Hydrogels sensitive to environmental stimuli exhibit promising prospects in functional applications. Changes in pH, temperature, and ion concentration are common in vivo or in different parts of human bodies; as such, hydrogels sensitive to those external triggers are ideal candidates for controlled drug delivery [232]. Additionally, hydrogels that can respond to molecules, light, pressure, moisture, and electrical signals also show promising potential in controlled delivery systems, biosensors, and bioseparation [233,234]. For the sensitive test, an equilibrium in the water content is introduced. For example, to test the temperature sensitivity, the prepared hydrogels are swollen to equilibrium in deionized water under a predetermined range of temperature. After 24 hours of incubation, the samples are removed, oven-dried, and weighed [224]. Similarly, the pH sensitivity can be evaluated by immersing the hydrogels in phosphate-buffered saline solution with different pH values. After equilibrium soaking, samples were wiped and then weighed [224,235]. The equilibrium water content of environmental-sensitive hydrogels is described based on the weight of swollen hydrogels at the equilibrium point and the weight of dried samples (3).

(3) $$\mathrm{Equilibrium}\;\mathrm{water}\;\mathrm{content}\;=\frac{m_{equilibrium}-m_{dry}}{m_{dry}}$$

where $m_{euqilibrium}$ and $m_{dry}$ represent the weight of swollen hydrogels at the equilibrium state and the weight of dried hydrogels, respectively.

Unconfined compression testing is the simplest method for comparing the mechanical characteristics of hydrogels. It is performed using a typical universal tensile testing equipment fitted with compression plates, a texture analyzer, or a similar setup [65]. Feki et al. used a texture analyzer to assess the texture profile of hydrogel based on chitosan and red algal polysaccharides [224]. Texture parameters, including springiness, hardness, stiffness, adhesiveness, and rupture force, were quantified. The analysis enables the determination of the mechanical characteristics of hydrogel samples and the extraction of the compressive elastic modulus from the initial slope of the compression test curve. This modulus indicates the viscoelasticity of the sample; moreover, cyclic compressive tests may be used to determine the elasticity of hydrogels and their long-term stability under stress [65]. Stiffness measurements also contribute to detecting the sensing behavior of live tissues since living cells are capable of feeling and responding to the stiffness of a substrate material [236].

Rheology is used to describe the deformation of hydrogels by establishing a relationship between deformation and the applied stress [210]. The rheological properties are performed on a rheometer with constant stress or constant shear mode, including oscillatory experiments (amplitude, time, frequency and temperature sweeps) and creep tests [223]. The properties are highly dependent on the structural types present in the system, such as association, entanglement, and crosslinks [210]. The dynamic mechanical/rheological characterization is helpful in understanding the formation mechanism of the hydrogels and, consequently, their possible applications. The dynamic viscoelastic functions, such as the dynamic shear storage modulus and loss modulus, were measured as a function of time, temperature, or angular frequency [237]. The storage modulus represents the elastic behavior, whereas the loss modulus indicates the viscous behavior of a sample. The investigation of these moduli elucidates the strength and network interactions of the prepared hydrogels [238]. 

The mesh size of hydrogel networks (the linear distance between two neighbouring crosslinks) is considered the most critical physicochemical property, especially for hydrogels applied in controlled delivery systems, because this feature controls the mass exchange between hydrogels and their external environments, then influencing the diffusion and release [239,240]. Apart from NMR, crioporosimetry, and release testing, the rheometer is another powerful tool that has been extensively used to estimate the mesh size of hydrogels [241,242,243]. The transparency of hydrogels is determined by the amount of light that passes through the gels. Hydrogel transparency varies depending on the treating temperature and coagulation bath used during the gelation process [244]. The degree of phase separation – liquid or solid change from one thermodynamic phase to two coexisting phases – has an impact on the transparency [245].

### 5.3. Biocompatibility, Biodegradability, Cytotoxicity, Injectability, and Drug Release Tests

Hydrogels as biomedical materials in controlled drug delivery, tissue engineering, and wound dressing have drawn considerable attention because of their complex reticulation and structural resemblance to the extracellular matrix [230]. To thoroughly investigate the biomedical applications, it is critical to develop frameworks that are biocompatible, biodegradable, and non-toxic. Moreover, ideal hydrogel candidates should also possess porous networks, various compositions and abilities allowing functionally tailored chemical modification, as well as be able to improve cell culture and new tissue formation [246,247]. Natural polymers such as algae-derived polysaccharides provide an opportunity to develop degradable hydrogels. To prepare a degradable hydrogel, the structure of the degradable segment should pair with specific enzymes. Moreover, the activity site of an enzyme should be located in the target area for the drug/cell administration [248]. 

Biocompatibility is the interaction between biomedical materials and the target tissues or systems of patients. Thus, biocompatibility evaluation is an important indicator of the safety of drug delivery systems and scaffolds. The assessment applied to polysaccharides-based hydrogels usually involves chemical analysis, tissue cultures, and animal tests [7,8,249,250,251,252,253,254,255]. However, risk-free drug delivery systems do not exist, so those tests aim to screen systems or materials with maximum benefits and minimum risks. Tissue cultures refer to using isolated cells to assess the cytotoxicity of materials, including qualitative and quantitative cytotoxicity tests. Qualitative tests are comprised of the direct contact procedure, the agar diffusion assay, and the minimum essential medium (MEM) elution assay, among which, the direct contact method is extensively used to evaluate the cytotoxicity of polysaccharides-based hydrogels used in the biomedical field [7,251,252]. Quantitative cytotoxicity evaluation shows higher priority than qualitative tests for the screening [256]. The evaluation usually involves MTT (3-(4,5-dimethyl-2-thiazolyl)-2,5-diphenyl-2H-tetrazoline bromide) and/or XTT (2,3-bis(2-methoxy-4nitro-5-sulphophenyl)-5-[phenylamine)carbonyl]-2H tetrazolium hydroxide) assays. Those two colorimetric methods measure the reduction of MTT and XTT caused by mitochondrial succinate dehydrogenase in living cells. The formation of azure formazan indicates enzymatic activities of the dehydrogenase and the cell vitality, i.e., the amount of formed azure formazan determines the number of living cells [250,253,254]. Regarding an in vivo biocompatibility study, mice were used and equally divided into an experimental group and a control group. Mice in the experimental group were injected with hydrogel solutions, while those in the control group were injected with a placebo [249]. Furthermore, a hemocompatibility test is required to evaluate the blood compactivity of hydrogel devices that contact blood directly [8,254,255]. The hemolysis testing method measures the damage to red blood cells caused by the samples, and the thrombus testing method detects the formation of blood coagulation [257].

Hydrogels presented as solutions at a temperature below that of the human body (sol-gel changing under heat) can be developed into injectable biomedical materials [5]. Injectable aqueous hydrogels containing active ingredients or in situ cells, are a promising therapeutic approach with minimal invasiveness and pain [258]. Therefore, the prepared hydrogels should guarantee to transform into a sol state before injection and start gelation after injection. The gelation time can also be recorded through the tube inversion method [258]. The gelation time was determined as the time when the mixture stopped flowing upon the tube inversion. Additionally, oscillatory experiments and creep tests of rheological characterization have been employed to evaluate their injectability and stability [8,223].

Hydrogels used as drug delivery carriers require a test to evaluate the drug release kinetics. The assessment includes investigations on in vitro drug release and release mechanisms. According to the target sites, the drug release behaviors and mechanisms as well as hydrogel swelling and degradation patterns, are all simulated in different physiological environments. The in vitro drug release is tested based on the encapsulating capacity, which can be calculated by comparing the amount of the drug in hydrogels with the amount of the drug added (4).

(4) $$Encapsulating\;capacity=\frac{M_{drug\;in\;hydroges}}{M_{drug\;added}}$$

where $M_{drug\;in\;hydroges}$ and $M_{drug\;added}$ represents the amount of the drug in hydrogels and the amount of the added drug, respectively.

Moreover, to determine the in vitro drug release mechanism of a certain drug from desired drug delivery systems, corresponding release data should be fitted to zero order, first order, Higuchi and Ritger-Preppas model [259]. For example, Cong et al. used an alginate-based hydrogel for controlling drug delivery via oral administration [260]. They tested the release behaviors and mechanism under simulated gastrointestinal conditions (i.e., simulated gastric fluid, simulated small intestinal fluid, and simulated colonic fluid). In the in vitro drug release test, emodin-loaded hydrogels in a dialysis bag were immersed in different simulated release mediums at predetermined time intervals. The obtained results were individually fitted to release models to explore the release mechanism. 

## 6. Techno-Economic Analysis of Polysaccharide-Based Hydrogels

Recently, researchers and engineers have been seeking opportunities to promote the value of algae by making the best use of bioactive compounds. The potential of seaweed-derived polysaccharides has been exhibited in hydrogel production at the industrial scale due to the enormous numbers and varieties of algae, their several eco-friendly inherent characteristics, and broad applications. Before the advent of the scale-up of hydrogel production from algae, it is significant to assess the technological and economic viability and profitability of seaweed polysaccharide-based hydrogels due to uncertainties in this emerging industry. 

A techno-economic analysis is regarded as a powerful decision-making tool involving techno-economic and sensitivity analyses, which has been widely used for production evaluation and market values estimate. The analysis usually begins with defining the scope of the problem and identifying the study’s inputs based on a market analysis. This step comprises all the input data needed to construct the techno-economic model in the second phase. Based on that information, economic calculations and technical calculations are performed, respectively, in which costs and revenues, as well as the performance metrics of the proposed project, are estimated. Finally, an evaluation, including investment analysis and performance analysis, is conducted according to the outcomes of calculations [261]. To build a well-balanced techno-economic analysis, researchers should estimate potentials and profitability, compare alternatives and make trade-offs between costs and performance while simultaneously introducing the sensitivity analysis to determine the capacity of systems to retain variation under current economic conditions. Many researchers and engineers have developed several ways and various parameters for measuring the overall techno-economic performance of existing or novel chemical processes; for instance, Herrera-Rodriguez et al. evaluated the industrial production of agar from red algae using the techno-economic sensitivity analysis [32]. However, there is a large knowledge gap in techno-economic analysis associated with seaweed polysaccharide-based hydrogel production. In spite of it all, researchers can obtain some ideas from articles related to the techno-economic analysis of other polysaccharide-based products or other hydrogels. 

Ruano et al. analyzed the economic and technological performance of chitosan-based hydrogels crosslinked through the interaction of hydrogen bonds [262]. Researchers simulated the four-step process of chitosan hydrogel formation and the consumption of raw chitosan is one ton per day and determined mass and energy balances, obtaining hydrogel yields on the basis of per kilogram of chitosan used. Regarding the economic assessment, Ruano and his coworkers evaluated capital costs, operating costs, economic profitability, and net present value for the production of 1 kg of hydrogels for a ten-year project in Colombia. The input parameters for economic assessment, such as utilities, reagents, and staff were obtained based on the average price in the Colombia context or from some manufacturers, such as Ensium or Alibaba International. For instance, the total raw materials cost was 284,000,000 USD/year, the total operating labor and maintenance cost was 63,560 USD/year, the total utility cost was 25,113.60 USD/year, etc. The results indicated that the largest contributor to the total production costs was raw materials, accounting for 92% of the total costs. The hydrogel production cost with the conditions set forth in this study was 3.023 USD/kg. Based on the estimated market price of chitosan-based hydrogels (3.3 USD/kg) and the project life (ten years), the net present value reached positive economic margins from the sixth year. Nevertheless, at the industrial level, the proposed assessment is incomplete due to the absence of algae cultivation and chitosan extraction; as such, researchers should consider covering those stages in their future work to demonstrate a complete agreement on the techno-economic feasibility. Furthermore, a comprehensive assessment integrating technical, economic, and uncertainties has not been fully addressed in this research; a techno-economic evaluation along with sensitivity analysis offers deeper insights into chemical process performance.

An industrial-scale assessment of a chemically assembled adsorptive hydrogel from process engineering and economic considerations and sensitivity analysis was performed [263]. The evaluation was based on actual practice related to the adsorptive hydrogel production in a pilot plant. From the technical aspects, the hydrogel production plant consisted of three parts–materials pre-treatment, hydrogen reactor, hydrogel processing, and relevant equipment specifications for a capacity of 3000 tons of hydrogel production per year were presented. Financially, the analysis included basic estimates, estimated costs, and sensitivity analysis, among which sensitivity analysis explored the impact of variation in selling prices and capital investment and annual production costs on profitability. Researchers estimated the total capital investment and the production cost were about 10,600,000 USD and 6,500,000 USD/year, respectively. In most industrial processes, the capital investment and production cost represent more than 50% of the total costs [264]. Furthermore, they assessed sales prices varied from 2.6 to 3.4 USD/Kg with an average selling price of 3 USD/kg. Under that scenario, sensitivity analysis used net present value, internal rate of return, and average simple return of return under different selling prices to demonstrate the profitability of the proposed project. Those profitability indicators could exceed 24,000,000 USD, 18%, and 23% at the assumed highest selling price [263]. As a result, the comprehensive techno-economic analysis strongly supported that the projected plant has promising feasibility prospects while simultaneously suggesting that the development of hydrogels with broad applicability would enhance the techno-economic viability. 

Meramo-Hurtado and González-Delgado applied techno-economic analysis to assess the production of chitosan-based adsorbents modified with titanium dioxide at the industrial scale [265]. In this study, they suggested using a step-wise method to evaluate innovative and existing chemical processes for the production of chitosan-based bio-adsorbents and select suitable design alternatives from a combination of the conventional economic evaluation method and techno-economic sensitivity analysis, which involves task definition by the knowledge of resources, products, and processes; data assembling of preliminary mass, energy balance, production yields, and flow diagram; and computer-assistant evaluation under techno-economic and sensitivity analyses. Scientists derived economic analysis models and data from the literature, chemical engineering magazines, vendors, and suppliers to determine and estimate equipment costs, installation costs, material and reagent costs, etc., in Colombia with a processing capacity of 680 t/year. As such, fixed capital investment, working capital investment, start-up costs, direct production costs, fixed charges, overhead, and general expenses were calculated, and then total capital investment, total operating cost, annualized fixed costs, and annualized operating costs were estimated. Researchers determined that the total capital investments of chitosan microbeads and titanium dioxide-modified chitosan microbeads were 6,009,282.06 USD and 29,627,718.49 USD, respectively, while the production costs were around 37,838,536.68 USD/year and 64,792,191.25 USD/year correspondingly. Moreover, they performed an economic analysis to evaluate the break-even point and then further determine profitable and marketable selling prices for both non-modified and modified chitosan microbeads. The assessment showed that the break-even points of those two processes were 58.80 USD/kg (non-modified) and 29.60 USD/kg (modified), respectively. This study fixed the start-point selling price for those two products to be 15% and 25% higher than the break-even point, as 64.60 USD/kg and 37.00 USD/kg. Those data enabled the sensitivity analysis, indicating that the non-modified chitosan microbead pross would be profitable with a net present value of 35,970,000 USD and an annual revenue of 4,380,000 USD, and the modified process would be an attractive alternative with a net present value of 135,160,000 USD and an annual revenue of 16,470,000 USD. The comparison exhibited that the modified method was a promising option for new businesses based on the green chemistry principles and the sustainable development. Moreover, they used sensitivity analysis to further evaluate uncertainties related to economic parameters, including the effect on the break-even point based on the production capacity, the effect on the on-stream efficiency by changing product selling price, the variation of raw material costs, profit after taxes, and return on investment. The far-reaching study revealed that compared with the chitosan microbead processing, the alternative of chitosan microbeads modified with titanium dioxide nanoparticles was in a better economic behavior under uncertainties and had a more competitive market price [265]. 

However, researchers and engineers have to brainstorm more parameters focusing on cost sensitivity in the actual practice. The identification of other important cost drivers in sensitivity analysis can enrich the assessment. For example, designing a hydrogel with chemical crosslinkers usually involves the removal of crosslinkers; this process requires a certain amount of inputs with cost sensitivity. The selection of a pH regulator may also cause extra manufacturing costs–for instance–using alkaline aqueous solutions instead of ammonia gas in chitosan-based hydrogel production results in the formation of silver oxide, which would affect the color and rigidity of the hydrogel [262]. 

The cultivation mode of seaweeds, extraction methods for target compounds, marketing aspects, logistics/transportation, packing and storage, among others, should be discussed in the economic analysis of seaweed polysaccharide-based hydrogel production. In conclusion, the most profitable option is determined by not only the economic potential acquired but also the response of the processes to the various changes according to the sensitivity analysis.

## 7. Application of Polysaccharide-Based Hydrogels

**Figure 24:**
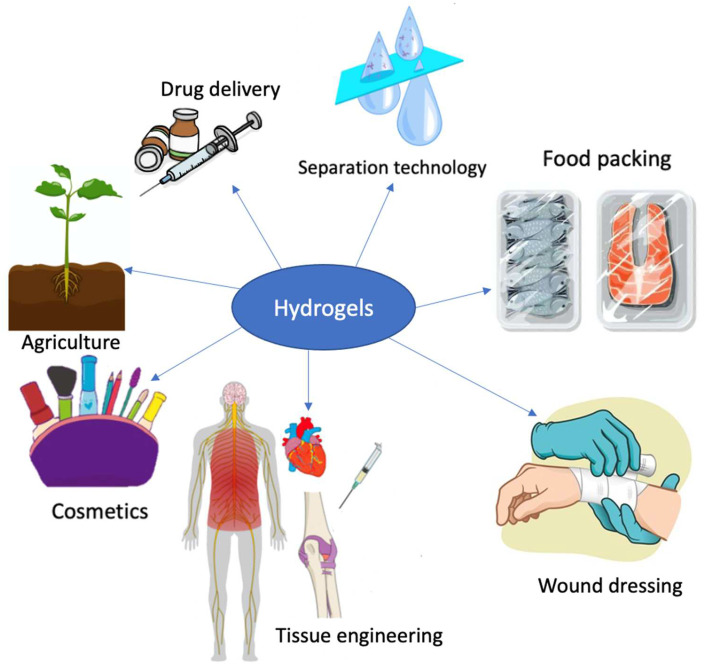
Applications of polysaccharides-based hydrogels.

Algal polysaccharides-based hydrogels have not only been widely applied to the biomedical field as drug delivery carriers, tissue engineering materials, and as wound dressings but have also emerged in food packing, separation technology, agriculture, and cosmetics (Figure 24). 

### 7.1. Drug Delivery

A considerable amount of active pharmaceutical ingredients has been discovered serving as therapeutics. However, due to the low bioavailability of therapeutic active compounds, they usually show poor activities in vivo and rapidly drop out of an effective range, requiring re-administration [2,266]. To solve the decreasing patient compliance and the increasing possibility of an overdose, controlled delivery systems as an alternative approach to regulate bioavailability are of significant attention.

A controlled drug delivery system is defined as the “formulation of a device that enables the introduction of therapeutic substances into the body and improves efficiency and safety by controlling the rate, time and place of drug release in the body” [267]. It aims to administrate active pharmaceutical ingredients to targeted sites at a specific rate and retain therapeutic benefits within the effective window for a certain period. However, it is hard to achieve that goal only using traditional drug delivery methods due to the lack of mechanisms to release active agents constantly [268]. As a result, controlled drug-delivery systems based on hydrogels have been designed to optimize the dosage in human bodies, achieving the regulation of costs, dosage frequency, and drug toxicity, and hence increasing the efficiency of drug delivery [1,2]. 

Due to their high swelling as well as excellent biocompatibility and biodegradability, algal polysaccharides-based hydrogels are more often used in drug delivery systems according to different delivery strategies, such as passive targeting, receptor-based targeting, and stimuli-responsive release [269]. For example, pH-sensitive drug delivery systems designed based on the variability of pH gradient along with the gastrointestinal tract have been proven to be effective in preventing drug degradation as well as improving drug absorption and controlled delivery. A novel controlled drug delivery system based on alginate hydrogel was designed [260]. The drug release tests revealed that the pH-sensitive alginate hydrogel could achieve sustained release and site-specific delivery for unstable or hydrophobic drugs. Bioavailability, which describes the ability of a drug or other substance to be absorbed and used by the body, can also be improved by drug-loading hydrogels. Nguyen et al. employed carrageenan and collagen composite hydrogels to enhance the stability and availability of allopurinol (a drug for gout and high levels of uric acid) [270]. They found thar the hydrogen bonds of the functional groups in allopurinol with the functional groups in carrageenan and fish scale collagen contributed to the bioavailability of allopurinol. 

Recently, researchers have also investigated pH-responsive drug-delivery systems based on fluorescent nanocellulose and agarose hydrogels [271], agarose hydrogel-based controlled drug delivery system with photothermal sensitivity [272], alginate/chitosan/carboxymethyl cellulose-based magnetic hydrogels for pH-sensitive drug delivery [273], pH-sensitive carrageenan-based hydrogel beads for allopurinol delivery [270], and pH-responsive alginate/kappa-carrageenan hydrogel beads for controlled protein release [274].

### 7.2. Wound Dressing

Wound healing involves four distinct stages: coagulation, inflammation, cell migration and proliferation, and remodeling. Normally, a healthy human body can support a wound healing process if no abnormalities in the skin’s function or massive fluid losses are observed [275]. The healing process can be sped up by providing suitable pH, humidity, oxygen pressure, and protection from microbial invasion. Hydrogel-based wound therapy method has been regarded as one of the most effective treatments. Hydrogels offer a biocompatible platform, sufficient moisture, and the ability to isolate microorganisms, hence achieving rapid healing for patients [230,276]. Polysaccharides-based hydrogels facilitate drug delivery through swelling in physiological fluids. Initially, the hydrogel interface experiences swelling, followed by the drug slowly dissolving in physiological fluids and diffusing from the hydrogel networks [226,277].

Polysaccharides-based hydrogels that adapt to specific physiological conditions are gaining growing attention for use in wound healing drug administration, among which alginate from brown algae is a candidate of potential. It can potentially promote wound healing by boosting angiogenesis, promoting cell migration, and decreasing the levels of proinflammatory cytokines at the wound site. Due to the network structure and hydrophilic nature, alginate hydrogels can absorb a large number of injured tissue fluids. Furthermore, they do not adhere to wound tissues, and their removal causes no further injury to the wound’s surface [230]. However, alginate hydrogels only provide a moist environment for wound healing; the lack of mechanical strength, angiogenesis, and antibacterial properties limits their application [129]. As such, researchers usually introduce substances that can enhance those properties during the synthesis of alginate hydrogels. Li and his coworkers designed a hydrogel based on sodium alginate and hardystonite bioceramic using double ion crosslinking (calcium ions and zinc ions), which exhibited great biocompatibility by creating a moist, antibacterial environment in a wound dressing [278]. Zhou et al. prepared novel sodium alginate-polyacrylamide hydrogels using divalent ion crosslinking (ions of copper, zinc, strontium, and calcium) [129]. They found zinc crosslinked hydrogels exhibited a series of improved properties–antibacterial activity, cell viability, mechanical strength, and wound closure ability–through encouraging fibroblast migration, vascularization, collagen deposition, and granulation tissue formation. Recently, alginate hydrogels with embedded zinc oxide nanoparticles have been designed for antibacterial purposes that are applicable for wound healing [279]. Researchers in this study observed that increasing zinc oxide concentration improved humidity retention in the hydrogels. In those three studies mentioned above, the addition of zinc or zinc oxide and hardystonite bioceramic serves as antibacterial components. Furthermore, some polysaccharides, such as chitosan, fucoidan, and chondroitin sulphate, naturally possess characteristics of hemostatic and bacterial and fungus inhibition [230]. To take advantage of the properties that chitosan has, Hu et al. combined alginate and modified chitosan (N-carboxymethyl chitosan) to prepare a hydrogel with epidermal growth factor payload using electrostatic interaction and divalent chelation [130]. The dual-crosslinked polysaccharide hydrogels were reported as a promising material that can promote cell proliferation and accelerate wound healing. Similarly, Ehterami and his coworkers employed alginate and chitosan to develop hydrogels containing Alpha-tocopherol (vitamin E) [280]. They evaluated the potential of this vitamin E-loaded hydrogel for wound healing in a rat model, revealing that the synthesized hydrogel with 400 IU vitamin E supported the highest wound closure. Moreover, the weight loss of 80% in 14 days showed that the prepared hydrogel is biodegradable. 

Hydrogels based on alginates have already been sold as commercial wound dressing products, such as Algicell™, AlgiSite M™, Comfeel Plus™, Kaltostat™, Sorbsan™, Tegagen™, SeaSorb®, etc. [281].

### 7.3. Tissue Engineering

Tissue engineering is a technique used to reconstruct organisms using artificial cellular scaffolds that resemble the extracellular matrix. Materials employed in tissue engineering should be non-toxic and biocompatible and have adequate mechanical strength in order to regenerate functional and useful tissues. These scaffolds can be cultivated in vitro followed by being transported to patients, or they can be injectable constructs containing cellular infiltration developing in vivo. Among the several biocompatible materials explored for tissue engineering, hydrogels have attracted considerable interest as their structures are similar to the natural extracellular matrix with adjustable viscoelasticity and high permeability to oxygen and nutrients [230]. Therefore, hydrogels composed of multiple functional components have been widely investigated in tissue engineering. 

As an important material for tissue engineering, hydrogels interact with the surroundings of live organisms, acting as biomimetic scaffolds, creating a humid and biocompatible environment for cell growth, controlling the release of active ingredients, and enabling long-term survival and activation of living cells [282,283]. Particularly, polysaccharides-based hydrogels are ideal materials as scaffolds for cell support due to their excellent biocompatibility, biodegradability, and low immunogenicity [282,284,285,286]. Furthermore, apart from their primary function as supporting materials in plants (cellulose) and energy sources in organisms (starch), polysaccharides have been reported to have a diverse range of pharmacological effects, including tissue repair and antibacterial, antiviral, antitumor, and immune system regulation properties [287]. 

The precondition for an applicable polysaccharide hydrogel scaffold that needs to be considered is the foreign-body response. By adjusting the natural chemical structure of polysaccharides and their derivatives, as well as the three-dimensional structure of polysaccharides-based hydrogels, the severity of foreign-body response can be relieved or managed. Alginate-based hydrogels are commonly used in living cell delivery since they exhibit mild stimulation to induce a foreign-body response in the host immune system, known as immunogenic; however, they are ineffective in preventing foreign-body response after long-term implantation [282]. Zhang et al. employed negatively charged alginate and positively charged poly(ethylene imine) developed an innovative hydrogel that can resist foreign-body reactions [288]. This neutrally charged hydrogel exhibited great antifouling properties, prevented capsule formation in vivo for at least three months, and significantly reduced macrophage migration to the interface between tissues and the implant. The reported modification altered the surface charge of the hydrogel, reducing protein adsorption and hindering the adhesion of proteins, cells, bacteria, and fresh blood on its surface [282,288]. Additionally, the hydrophilicity and the size and shape of polysaccharides-based hydrogels also play a critical role in regulating protein adsorption and inhibiting the foreign-body reaction [282]. 

Due to the complexity of biological environments, such as in cell proliferation and differentiation and tissue repair, injectable hydrogels are more suited for their intended applications in contrast to typical bulky hydrogels [289]. They can be injected as a liquid and crosslinked to form a gel phase with the help of specific stimuli, and they can also be injected in a partially crosslinked gel form. Injectable hydrogels have demonstrated significant potential for local administration of therapeutic cytokines, recombinant proteins, and secretions of living cells such as exosomes [290]. Because alginates showed promising results in the treatment of myocardial infarction, they have become the very first injectable materials in clinical trials in the myocardial infarction treatment [291]. Researchers prepared calcium crosslinked alginate hydrogels to determine whether they can be delivered effectively into the infarcted myocardium by intracoronary injection. In a recent study, Mayfield and her coworkers also developed agarose-based hydrogel capsules to encapsulate cardiac stem cells [292]. The cells were injected into mice with infarcted hearts; as a result, the researchers found that the agarose hydrogel encapsulated cardiac stem cells enhanced cardiac repair and blood vessel formation.

### 7.4. Agriculture

Based on the primary study of controlled release systems in drug delivery, the application of such systems is extended to agriculture, such as using environmentally friendly, cost-effective, biodegradable platforms to carry agrochemicals for the controlled release [293]. Polysaccharides-based hydrogels are the most recommended carrier vehicles because of their biodegradability, non-toxicity, strong loading capacity, and hydrophilic properties [294]. By modifying some properties (e.g., moisture, pH, temperature, ion concentration, etc.), agrochemicals can be timed-release into the soil and retain their concentrations in optimal ranges. That avoids overdose and unexpected spending and also reduces the adverse environmental impacts in the agricultural practice [295]. Therefore, polysaccharide-based, biodegradable hydrogels have recently attracted increasing interest in the agriculture sector.

The most common agricultural application of polysaccharides-based hydrogels is the controlled release of agrochemicals, such as fertilizers, pesticides, and herbicides. It has been reported that some of those substances pose significant potential risks to human health [296]. Additionally, the hydrogels are also used as soil conditioners for erosion control. Conditioners have aided in increasing the water retention capacity in the soil, reducing the irrigation frequency, lowering expenses, and enhancing yields in the fields [297]. 

Bortolin et al. synthesized hydrogels using poly(acrylamide) and methylcellulose to explore their applicability as potential delivery vehicles for the controlled release of ammonium sulphate and potassium phosphate fertilizers [293]. Researchers pointed out that methylcellulose increased the loading quantities of fertilizers and extended the time and amounts released, which was strongly affected by the hydrophilic properties of the prepared hydrogels. Işıklan encapsulated pesticide carbaryl in alginate hydrogel beads to study the controlled release formulations that could effectively reduce the environmental impact of pesticides [9]. The hydrogel beads were designed by the ionotropic crosslinking of sodium alginate with calcium and nickel ions. It was observed that carbaryl releasing from the calcium alginate hydrogel beads was slower than that from nickel alginate hydrogel beads. Moreover, as the crosslinker and sodium alginate concentration, as well as the ratio of carbaryl to sodium alginate, increased, the releasing rate would decrease in both hydrogel beads.

Due to the severe water resource crisis on the planet, it is necessary to enhance the water-holding capacity in the soil and achieve water-saving agriculture. Hydrogels based on natural polymers such as polysaccharides have emerged in the agriculture sector as soil conditioners. Cellulose-based, starch-based, and cellulose/starch composite hydrogels were subject to overcome the scarcity of water in tomato (*Solanum lycopersicum*) [10]. Researchers found that when using cellulose-based, starch-based, and cellulose/starch composite hydrogels in the experimental trials with water at 50 % of the field capacity, the increase in fruit number reached 1.02, 1.45, and 1.69-fold compared to the control field. As a result, the irrigation frequency could reduce by 40% to maintain the productivity that was created by full irrigation without using polysaccharides hydrogels-based controlled release systems. Several recent articles in the agriculture field using starch-based hydrogels as soil conditioners have also been reported [298,299].

### 7.5. Separation Technology

The use of low-cost, biodegradable adsorbents is an effective strategy to minimize the environmental impact caused by human activities. Furthermore, in comparison with separation techniques, such as precipitation, electrodialysis, and nanofiltration, adsorption provides several advantages, including high recovery efficiency, simple operation, and lower energy demand [300,301,302]. Therefore, researchers have investigated a variety of biodegradable and effective hydrogel adsorbents derived from natural sources to remove contaminants, such as heavy metals, dyes, and agrochemicals, from aqueous solutions under different operation conditions [294,300,303,304,305].

Heavy metal contamination of different water supplies has become a widely discussed topic because of the detrimental effect on human beings, animals, and plants. Many industries, such as mining, metal plating, electric device manufacturing, etc., are important contributors to heavy metal pollution. Hydrogels comprising amide, amine, carboxylic acid, and ammonium groups have been reported to have the ability to bind metal ions [306]. To improve the adsorption capacity, acidic resistance, and mechanical strength, as well as design a reusable hydrogel adsorbent, Li and Bai studied copper adsorption with chitosan-cellulose hydrogel beads [305]. Moreover, inspired by the metal adsorption characteristics of brown algae–lithium can be adsorbed by carboxylic acid and phosphonate groups in brown algae–alginate hydrogels incorporated with phosphonate metal-organic framework were fabricated. The proposed alginate hydrogels were reusable after being washed with ethanol or deionized water [300]. 

Agrochemicals include a diverse range of chemicals, some of which are known as poisons or toxins for human health. Over-application of them contaminates surface water and groundwater. Conventional drinking water treatment methods have been demonstrated to be ineffective in removing pesticides, such as metolachlor and terbuthylazine [307]. As such, adsorption is a frequently used approach to removing toxic agrochemicals [308]. Except for broadly used membranes, researchers started to investigate the adsorption of agrochemicals using hydrogels. Aouada et al. studied the application of poly(acrylamide) and methylcellulose hydrogels as a novel adsorbent material for the removal of pesticide paraquat from an aqueous solution. The hydrogels were prepared through a free-radical polymerization method [294].

Dyes are also a significant pollutant that occurs downstream of printing houses, clothing factories, food processing industries, and hair salons. Although dyes are not extremely dangerous, they can still produce specific adverse effects in humans, including increased heartrate, vomiting, shock, cyanosis, jaundice, quadriplegia, and tissue necrosis. Manufacturing sectors discharge effluents into rivers and lakes, altering their biological life. To effectively remove dyes by adsorption process, Duman et al. developed Agar/kappa-carrageenan composite hydrogel adsorbents using the free radical polymerization method for the removal of methylene blue dye [11]. Hydrogels composed of cellulose were also synthesized and used to remove methylene blue from an aqueous solution. To increase the purification efficiency, titanium dioxide nanoparticles were encapsulated into cellulose nanofibers [303]. Notably, researchers treated prepared kappa-carrageenan-based hydrogels with potassium ions to obtain hydrogels with magnetic properties, which showed selectivity to cationic dyes [156].

### 7.6. Food Packaging

Developing biodegradable packaging from renewable resources is critical for resolving environmental issues and energy crises associated with the usage of petroleum-based plastics; furthermore, paper-based packaging performs poorly when in contact with gas/oil and requires technological innovations [13,14]. As a result, edible food packaging made from polysaccharides has garnered considerable attention due to its biodegradability, biocompatibility, safety, and abundant sources. The packaging based on polysaccharides hydrogel films has been reported to feature transparency and superior mechanical properties; meanwhile, they can also be combined with other active materials to impart additional characteristics to the hydrogel, such as antibacterial and antioxidant properties [14,309]. Compared with conventional synthetic polymers, polysaccharides-based hydrogel films demonstrate similar tensile strength. Furthermore, it has been demonstrated that such films can extend the shelf-life of ready-to-eat foods, effectively prohibit gas transference, and possess e a poor water vapor permeability [309].

Şen et al. developed a naturally antimicrobial hybrid hydrogel based on cationic starch and sodium alginate via electrostatic interactions [134]. The produced food packaging hydrogels were reported to have good thermal, antimicrobial, and surface properties, and they could be used as food packaging materials in many industries.

Dai et al. investigated a self-assembled polysaccharide hydrogel film for paper-based food packing [13]. The introduction of TEMPO-oxidized cellulose nanofibers/cationic guar gum hydrogel film significantly modified the mechanical and barrier properties of the paper through a layer-by-layer deposition on paper. Additionally, the packaging test indicated the hydrogel film had the oil-repellent property while simultaneously could maintain the freshness of foods. 

Benavides et al. designed an alginate-based hydrogel film through mechanical stirring and ionic interaction, among which the ionic interaction crosslinking indicated alginates could react with di- and tri-valent cations to form hydrogels [310]. The calcium ions form an internal crosslinking between 1, 4-β-d-mannuronic acid and α-L-guluronic acid, resulting in a three-dimensional network. The method of crosslinking with polyvalent cations has been used to increase the water barrier, mechanical resistance, cohesiveness, antibacterial property, and stiffness of polymeric materials, as well as to postpone the release of some substances. Moreover, calcium that is released slowly in an acid solution aids in the formation of homogenous matrices. 

Wu et al. prepared an edible hydrogel film with chitosan and agarose for food packaging [14]. Notably, the proposed hydrogel film incorporated anthocyanin as the pH-responsive indicator. Through observing the color change of anthocyanin caused by the total volatile basic-nitrogen released by the enzyme-catalyzed decomposition of fish, the hydrogel film is applied to monitor the spoilage of fish.

Hence, polysaccharides-based hydrogels have the potential to enable the development of innovative biodegradable food packaging and to serve as an alternative to synthetic polymers in food science. That might help address the environmental issue of waste accumulation caused by non-biodegradable petroleum-based plastics.

### 7.7. Cosmetics and Personal Care Products

Polysaccharides-based hydrogels are also applied in some emerging industries, such as in cosmetics and personal care products. With the development of economics and dermatology, people realize cosmetics and personal care products can function as extra barriers to protecting their bodies from various environmental influences and provide benefits like moisturizing at the same time.

Hyaluronic acid is a widely studied linear polysaccharide in the cosmetic industry, with water-soluble and highly viscoelastic properties [15]. It is commonly used as topical cream/lotion/serum or dermal filler to improve skin hydration and elasticity, restore skin volume, and reduce wrinkles, leading to smoother skin [311]. As for the application in dermis injection, hyaluronic acids mimic the cellular components effectively and guarantee safety and tolerability for patients [312,313]. However, hyaluronic acid dermal filler usually turnovers rapidly within tissues due to the hyaluronidase enzymes. To increase the half-life of hyaluronic acids, several modification methods have been investigated [314]. Those methods were mostly used to block the access of the hyaluronidases to the polysaccharide-based hydrogels by crosslinking hyaluronic acids; as such, the three-dimensional, hyaluronic acid-based hydrogels were more resistant to enzymatic degradation than original hyaluronic acids because of the formation of covalent bonds between the hyaluronic acid molecules and the crosslinkers [315,316,317]. Thus, hyaluronic acid-based hydrogels have been widely used for a variety of skin problems, such as wrinkles, nasolabial folds, collagen loss [312,313,318], and hair care products [319]. Hydrogels based on brown algae-derived alginate were regarded as alternative submucosal injection materials to hyaluronic acids, with the properties of durability, safety, and cost-effectiveness [320]. Alginate-based polymer microbeads were also prepared as environmentally friendly cosmetic additives to replace the non-biodegradable microparticles in exfoliation products [321,322]. The study showed that alginate polymer microbeads did not cause irritation, redness, itching, or dryness [322]. Carrageenan-based hydrogels can be considered for cosmetic applications as well [323]. The hydrogels were designed in the form of cosmetic pads/facial masks, loading active ingredients including hyaluronic acids, panthenol, tocopherol, etc. Some patents involve carrageenan-based hydrogels with excellent adhesion, moisturizing effect, gelling performance, and rheological properties for applications in facial masks or partial patches [324,325]. Currently, algal polysaccharide-based hydrogels for cosmetics and skincare products are not yet very widespread; but their properties are similar to those of hyaluronic acids and chitosan, indicating the potential for the future market. 

Similarly to drug delivery systems, Johnson & Johnson published a patent that uses hydroxyethyl cellulose/hydroxypropyl cellulose crosspolymer porous microspheres to entrap tretinoin for the topical treatment of acne [326]. That suggests polysaccharides-based hydrogels are also suitable for active ingredient encapsulation in skincare products. Some active ingredients, such as retinoids, vitamin C, peptides, antioxidants, and sun filters, are unstable and sensitive to light, temperature, pH, and oxidation. When exposed to an undesired environment, those substances may degrade [327]. Therefore, hydrogels, especially polysaccharides-based hydrogels, are ideal for the encapsulation of active ingredients for cosmetic products. According to specific applications, the function of hydrogels can be custom designed. They can be time-released, stimuli-responsive, and/or degradable.

On the other hand, superabsorbent hydrogels with remarkable water absorption ability attracted increasing interest as diapers, adult incontinence products, feminine hygiene products, and other similar personal care products. Compared with traditional materials such as cotton, cloth, paper wadding, and cellulose fibre, the newly designed superabsorbent polymers based on polysaccharides appear to have higher absorption efficiency and better recyclability [16]. Erizal et al. used starch, acrylamide, and acrylic acid, crosslinked with gamma radiation to design fast swelling superabsorbent hydrogels [328]. Furthermore, biodegradable superabsorbent hydrogels were prepared using hydroxyethylcellulose and sodium carboxymethylcellulose crosslinked with divinylsulfone [329]. In 2004, Groupe Lysac Inc. patented a superabsorbent polymer based on carboxymethylcellulose and/or gums as novel biodegradable diapers [330]. However, polysaccharide-based superabsorbent hydrogels used in hygiene products are still in their early stages.

## 8. Conclusions and Future Prospects

The exploration of value-added compounds to promote the valorization of waste algal biomasses, especially those involving environmental concerns (e.g., algal bloom and eutrophication), offers a new perspective on integrated environmental management. Moreover, the development of commercial algae production not only contributes to carbon dioxide fixation, photosynthesis with oxygen generation, and better water quality but also extends to manufacturing value-added compounds for a variety of applications. Among those valuable compounds, polysaccharides have attracted increasing interest in high added-value chain of biomaterials due to their potential for biomass valorization. This review presents the extraction of algal polysaccharides, relevant characterization methods, the recent progress in the preparation of algal polysaccharides-based hydrogels, as well as their potential applications in biomedical, agricultural, environmental, and cosmetical sectors over the past decade.

Algal polysaccharide extraction mainly focuses on conventional, bench-level methods; as such, it is necessary to establish novel, green, and cost-effective methods with higher yields, better purity, and superior properties. Moreover, due to the presence of numerous algae species, various polysaccharides with unknown compositions and properties remain unexplored. To support the growth and expansion of industries based on algal polysaccharide hydrogels, polysaccharide extraction is expected to advance further and innovate in the development of scalable recovery processes. 

Hydrogels based on polysaccharides have unique properties such as high-water retention capacity, biocompatibility, biodegradability, and non-toxicity, making them ideal for various applications. Due to their adjustable structure and networked morphology, polysaccharides-based hydrogels present significant benefits, such as environmental sensitivity and controlled delivery. We reviewed the physical and chemical crosslinking approaches for algal polysaccharides-based hydrogels. Currently, because of their primary applications as biomedical materials or platforms, physical crosslinking methods, especially those based on electrostatic interactions, are widely used. 

Despite the fact that hydrogels derived from algal polysaccharides have great promise as biomaterials, some polysaccharides, such as porphyran and laminarin, are currently underexploited according to our findings. Moreover, there are still some challenges that should be considered during the hydrogel preparation, including: (1) the introduction of poisonous reagents or crosslinkers; (2) poor strength or uncontrollable properties of single-component hydrogels or single network structure; and (3) stimuli-responsive hydrogels with precise drugs/substances release at targeted sites and long-term stability.

For future studies, researchers should pay more attention to investigating green, low energy consumption hydrogel synthesis using non-toxic crosslinkers and solvents. The incorporation of multiple crosslinking strategies or a design of multiple structure networks could be a future direction to prepare polysaccharides-based hydrogels with enhanced strength and properties. As mentioned, there is a lack of techno-economic analysis of hydrogels based on algal polysaccharides, which covers all the stages related to algae growth and harvest, polysaccharides extraction and purification, hydrogel preparation, and/or applications. The blank in this area can also be a direction for future study. Moreover, new promising applications of algal polysaccharides-based hydrogels should be further studied. For example, hydrogels with antibacterial characteristics (e.g., algal polysaccharides/chitosan composite hydrogel, or zinc/titanium integrated algal polysaccharides-based hydrogels) as surface materials of infrastructure and public facilities, or solid hydrogels as conductive electronic devices, or hydrogels as vehicles to carry effective skincare ingredients, such as retinol, hyaluronic acid, panthenol, and vitamin C, for the purpose of sustained release and irritation relief. 

## Data Availability

Not applicable.
